# Design, Synthesis, and Cytotoxicity of Perbutyrylated Glycosides of 4β-Triazolopodophyllotoxin Derivatives

**DOI:** 10.3390/molecules20023255

**Published:** 2015-02-16

**Authors:** Cheng-Ting Zi, Zhen-Hua Liu, Gen-Tao Li, Yan Li, Jun Zhou, Zhong-Tao Ding, Jiang-Miao Hu, Zi-Hua Jiang

**Affiliations:** 1 Key Laboratory of Medicinal Chemistry for Nature Resource, Ministry of Education, School of Chemical Science and Technology, Yunnan University, Kunming 650091, China; E-Mail: zict@sina.cn; 2 State Key Laboratory of Phytochemistry and Plant Resources in West China, Kunming Institute of Botany, Chinese Academy of Sciences, Kunming 650201, China; E-Mails: liuzhenhua@mail.kib.ac.cn (Z.-H.L.); ligentao@mail.kib.ac.cn (G.-T.L.); liyanb@mail.kib.ac.cn (Y.L.); jzhou@mail.kib.ac.cn (J.Z.); 3 Department of Chemistry, Lakehead University, 955 Oliver Road, Thunder Bay, ON P7B 5E1, Canada

**Keywords:** podophyllotoxin, glycosylated, 4β-triazole, CuAAC reaction, antitumor, synthesis

## Abstract

A series of novel perbutyrylated glycosides of 4β-triazolopodophyllotoxin derivatives were synthesized by utilizing the copper-catalyzed azide-alkyne cycloaddition (CuAAC) reaction. Evaluation of cytotoxicity against a panel of five human cancer cell lines (HL-60, SMMC-7721, A-549, MCF-7, SW480) using the MTT assay shows that some of these glycosylated derivatives have good anticancer activity. Among the synthesized compounds, compound **21a** shows the highest activity, with IC_50_ values ranging from 0.49 to 6.70 μM, which is more potent than the control drugs etoposide and cisplatin. Compound **21a** is characterized by a perbutyrylated α-D(+)-galactosyl residue, the absence of an additional linking spacer between the sugar residue and the triazole ring, as well as a 4'-OH group on the E ring of the podophyllotoxin scaffold.

## 1. Introduction

**Figure 1:**
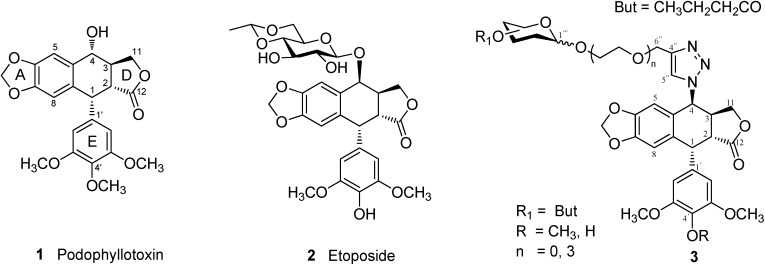
Structures of podophyllotoxin (**1**), etoposide (**2**) and podophyllotoxin derivatives (**3**).

Podophyllotoxin (**1**, Figure 1), a well-known naturally occurring aryltetralin lignan extracted from the roots of *Podophyllun peltatum*, has been known to inhibit the assembly of tubulin into microtubules through tubulin binding, but the high toxicity of podophyllotoxin has limited its application as a drug in cancer chemotherapy [1,2,3,4]. The potent anticancer activity of **1** has led to extensive structural modifications for the discovery and development of new anticancer agents. Etoposide (**2**, Figure 1) [5] is a semisynthetic glucosidic cyclic acetal of podophyllotoxin which is in clinical use as an antineoplastic agent against various cancers, including small-cell lung cancer, non-Hodgkin’s lymphoma, leukemia, Kaposi’s sarcoma, neuroblastoma and soft tissue sarcoma [3,6,7,8,9,10,11,12]. However, the therapeutic use of **2** is often overcome by the problems of drug resistance, myelo-suppression and poor oral solubility. In order to overcome drug resistance and improve topoisomerase II inhibition, various structure modifications of podophyllotoxin have been made [13,14], novel dimeric podophyllotoxins obtained by condensation of thiocolchicine and/or podophyllotoxin with six different dicarboxylic acids, having a marked ability to inhibit the polymerization of tubulin *in vitro* and the spacer unit was found to have a significant effect on biological activity [15]. According to structure-activity relationship (SAR) studies, 4*^′^*-demethylation, 4-epimerization, *trans*-lactone D ring with 2α, 3β configuration and free rotation of ring E were essential to maintain the anticancer activity of podophyllotoxin derivatives as topoisomerase-II inhibitors [16,17]. Studies have also demonstrated that substitution at C-4 is tolerable to significant structural diversification.

Traditional cancer chemotherapy is often accompanied by systemic toxicity to the patient, therefore the development of new antitumor drugs with increased selectivity and reduced toxicity is highly desirable. Recently, antibody-drug conjugates (ADCs) that use antibodies to deliver a potent cytotoxic compound selectively to tumor cells were approved for cancer therapy: CD30-targeting brentuximab vedotin for use in Hodgkin lymphoma and anaplastic large cell lymphoma (ALCL), and HER2-targeting ado-trastuzumab emtansine (T-DM1) for use in metastatic breast cancer [18]. Carbon nanomaterials are a source of materials that show unique biological applications for their π-electron cloud and structures. Species such as carbon nanotubes (CNTs), fullerenes, graphenes, carbon nanoparticles, nanodiamonds, carbon nanohorns and carbon nanocaps are common in the formulations of these nanomaterials as biosensors, imaging probes, drug and gene delivery systems, and nanomedicine [19]. By combination with other materials, the nanoarchitectures of nanocarbons can be formed into structures of different dimensions and properties for biological applications, especially cell growth, sensing, and control [20].

In recent years, the preparation of glycoconjugates of small molecule anticancer drugs has become an attractive strategy in order to improve drug efficacy. The clinically widely prescribed anticancer drug etoposide (**2**) is a β-d-glucopyranoside of 4'-demethylepipodophyllotoxin [21,22,23]. The anticancer activity of other types of podophyllotoxin glycosides, e.g., α-glucopyranoside, α*/*β-galactopyranoside, α*/*β-mannopyranoside, *etc*., has not been well studied. In our previous study [24], we reported 4β-triazole-linked glucose podophyllotoxin conjugates as a new class of antitumor compound; it was found that podophyllotoxin derivatives with a perbutyrylated glucose residue showed high activity. Reported here are the chemical synthesis of a series of perbutyrylated glycosides (D-Gal/D-Man/D-Xyl) of 4β-triazolopodophyllotoxin derivatives (**3**, Figure 1) conjugated with a specific monosaccharide residue and their *in vitro* anticancer activity against five human cancer cell lines, including HL-60 (leukemia), SMMC-7721 (hepatoma), A-549 (lung cancer), MCF-7 (breast cancer), and SW480 (colon cancer).

## 2. Results and Discussion

### 2.1. Chemical Synthesis

**Scheme 1:**
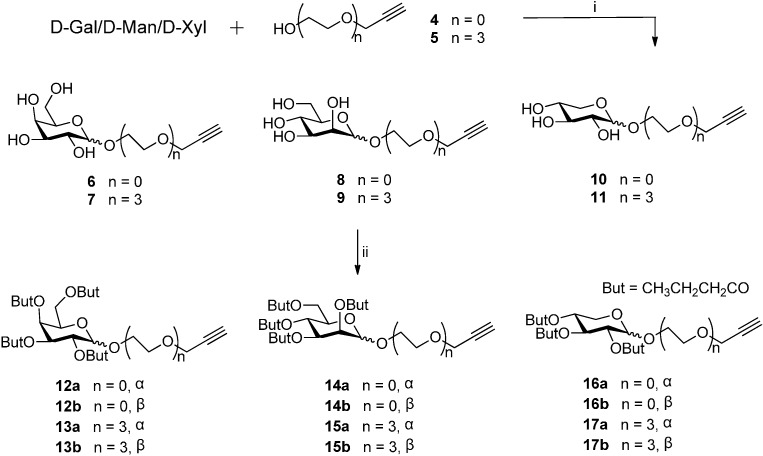
Synthesis of glucosylated terminal alkynes.

Since the 1,2,3-triazole ring moiety is a widespread functional group in drugs [25,26], the click reaction of copper-catalyzed azide-alkyne cycloaddition (CuAAC) has been widely used to covalently link two molecular fragments between a terminal alkyne and an azide to generate substituted 1,2,3-triazoles [27,28]. To facilitate the coupling of the sugar residue with the podophyllotoxin scaffold, a group of glycosylated terminal alkynes **12a/b**–**17a/b** have been prepared (Scheme 1). Fischer type glycosylation of D(+)-galactose, D(+)-mannose, or D(+)-xylose with propargyl alcohol **4** or its derivative **5** containing three ethyleneglycol units [29] in the presence of H_2_SO_4_-silica as a catalyst afforded the desired propargyl glycosides **6**–**11** as α/β mixtures in 69%–75% yield [30]. Compounds **6**–**11** were perbutyrylated with butyric anhydride and pyridine [31] to give the perbutyrylated glycosylated terminal alkynes **12a/b**–**17a/b**, in 89%–96% yield. In each case the α/β mixture was separated to give both the α- and β-anomer in pure form.

**Scheme 2:**
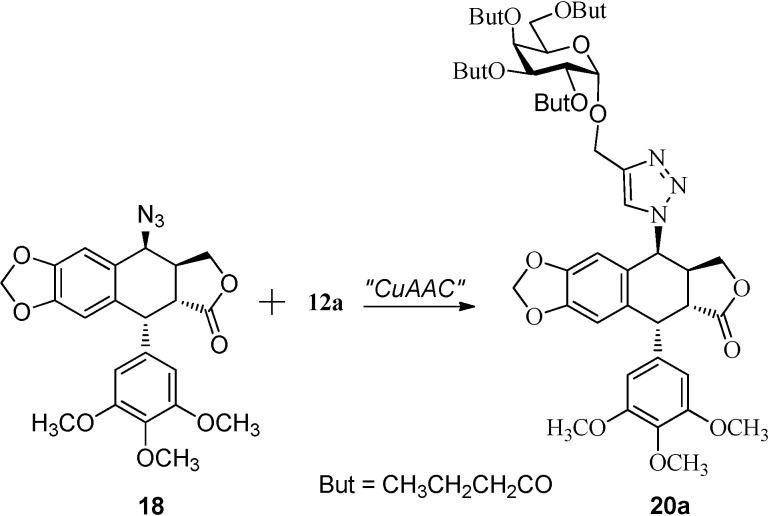
Click-chemistry strategy for the synthesis of the 1,2,3-triazole derivative **20a**.

Click chemistry involves a terminal alkyne and an azide that undergo a copper-catalyzed [3+2]-cycloaddition to generate a triazole ring [27,32]. There have been numerous reports documenting the best reaction conditions for this cycloaddition reaction [32,33]. It appears that the type of catalyst (copper species), the additive, the solvent, and the reaction time can all affect the yield of this addition reaction. We did a quick screening for the reaction conditions that would work best for our substrates. Thus alkyne **12a** was reacted with 4β-azidopodophyllotoxin **18** [24,34] under different reaction conditions to give the 1,2,3-triazole derivative **20a** (Scheme 2). The reaction conditions and the respective yields are listed in Table 1.

*Reagents and reaction conditions*: (**i**): cat. H_2_SO_4_-silica, 65 °C, 69%–75%. (**ii**): *n*-butyric anhydride, pyridine, 16 h, 0 °C, 89%–96%.

**Table 1:** Screening of the reaction condition for the CuAAC reaction between 4β-azido-podophyllotoxin (**18**) and the glycosylated terminal alkyne (**12a**).

Entry	Catalyst	Additive	Solvent	t (h)	Yield (%)
1	CuSO_4_·5H_2_O	Sodium l-Ascorbate	*t*-BuOH/H_2_O (1:1)	2	70
2	CuSO_4_·5H_2_O	Sodium l-Ascorbate	*t*-BuOH/H_2_O (1:2)	2	90
3	CuSO_4_·5H_2_O	Sodium l-Ascorbate	DMF/H_2_O (3:1)	2	63
4	CuSO_4_·5H_2_O	Sodium l-Ascorbate	DMSO/H_2_O (1:1)	2	80
5	CuSO_4_·5H_2_O	Sodium l-Ascorbate	*t*-BuOH	2	nr ^a^
6	Cu(OAc)_2_	Sodium l-Ascorbate	*t*-BuOH/H_2_O (1:2)	2	67
7	Cu(OAc)_2_	Sodium l-Ascorbate	*t*-BuOH/H_2_O (1:2)	31	87
8	CuI	None	MeCN	12	60
9	CuI	None	*t*-BuOH/H_2_O (1:2)	12	15
10	CuI	None	DMSO/H_2_O (9:1)	12	63

Note: ^a^ nr: no reaction.

As can be seen in Table 1, the reaction occurred with different solvents in the presence of CuSO_4_·5H_2_O and sodium L-ascorbate within 2 h (Entries 1–4). It is found that *t*-BuOH/H_2_O (1:2) as the solvent provided the highest yield. No transformation occurred in the presence of *t*-BuOH alone as the solvent (Entry 5). Using the combination of Cu(OAc)_2_ and sodium L-ascorbate as the source of Cu(I) species [35], the reaction time can affect the yield significantly (Entries 6,7). In the case of CuI-catalyzed reactions [32,33], the solvent was also found to influence the reaction rate (Entries 8–9); however, the reaction yield was not further improved compared to CuSO_4_·5H_2_O/sodium L-ascorbate system (Entries 1–4). Subsequently, CuSO_4_·5H_2_O/sodium L-ascorbate with *t*-BuOH/H_2_O (1:2) as the solvent and the reaction time of 2 h (Entry 2) was chose as the condition for the CuAAC reaction of all substrates reported herein.

**Scheme 3:**
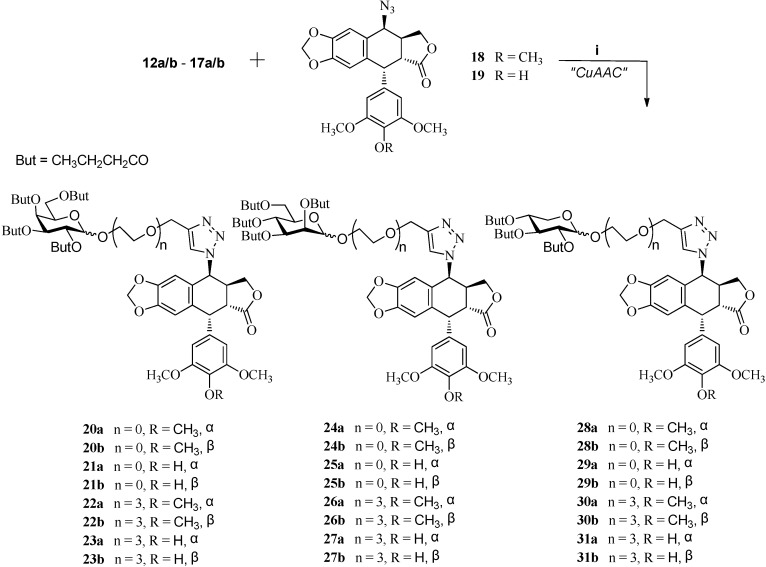
Click-chemistry strategy for the synthesis of 4β-triazole-podophyllotoxin derivatives.

The azides **18** and **19** [24,34] were allowed to react with the above terminal alkynes (**12a/b**–**17a/b**) in the presence of CuSO_4_·5H_2_O, sodium ascorbate in *t*-butyl alcohol and water (1:2) at room temperature to give glycosylated 4β-triazolopodophyllotoxin derivatives **20a/b**–**31a/b** in excellent yield (Scheme 3).

*Reagents and conditions*: (**i**): CuSO_4_·5H_2_O, sodium ascorbate, *t-*BuOH-H_2_O (1:2), 2 h, rt. 82%–92%.

All the products were characterized by ^1^H-NMR, ^13^C-NMR, ESI-MS, and HRESI-MS. In the ^1^H-NMR spectra, the formation of the podophyllotoxin triazoles was confirmed by the resonance of the C^5"^-H signal (δ7.72–8.33 ppm) of the triazole ring in the aromatic region, which was further supported by two characteristic carbon signals at around 123 ppm and 126 ppm in the ^13^C-NMR spectra. The configuration at the C-4 position for target compounds **20a/b**–**31a/b** was confirmed based on the *J*_3,4_ coupling constant, which is typically < 5.0 Hz for 4β-substituted compounds due to a *cis* relationship between H-3 and H-4 [36]. ESI-MS and HRESI-MS of all compounds showed the [M+Na]^+^ or [M+H]^+^ adduct as the molecular ion.

**Figure 2:**
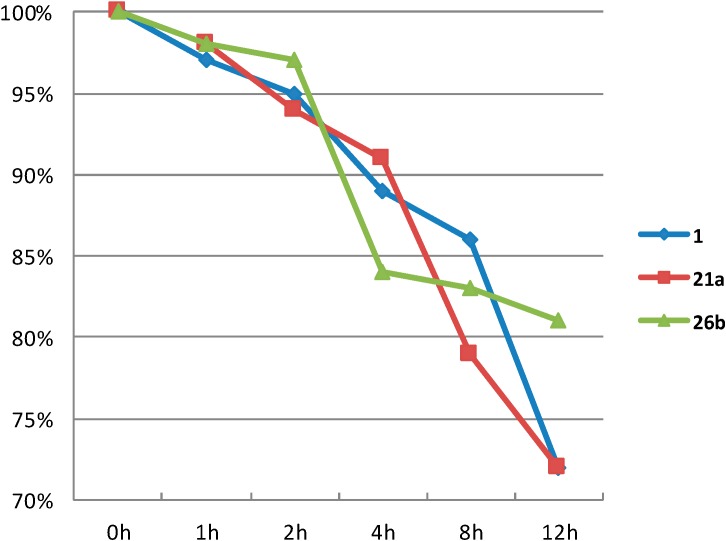
Chemical stability investigation of compounds **1**, **21a** and **26b**.

Two representative compounds (**21a** and **26b**) were selected for investigation of the chemical stability in aqueous phase in comparison of podophillotoxin (**1**). The results indicate that compounds **21a** and **26b** exhibit better chemical stability under the specific conditions (37 °C, pH = 7.0, Figure 2). Obviously, compound **26b** is the most stable one, and having the appropriate length of the linking spacer between the sugar and triazole ring and 4'-OCH_3_ on the E ring improved the chemical stability of podophillotoxin. These improvements make them much more drug-like than the natural parent podophillotoxin (**1**), and would be promising for the future further development.

### 2.2. Evaluation of Biological Activity

All the perbutyrylated glycosides of 4β-triazole-podophyllotoxin derivatives **20a/b**–**31a/b** were tested for their anticancer activity against five human cancer cell lines, including HL-60 (leukemia), SMMC-7721 (hepatoma), A-549 (lung cancer), MCF-7 (breast cancer), and SW480 (colon cancer). Etoposide (**2**) and cisplatin were taken as reference compounds. The screening procedure was based on the standard MTT method [37], and the anticancer activity data are presented in Table 2. Among these compounds **21a** shows the most active inhibition against all five cancer cell lines tested, with IC_50_ values ranging from 0.49 to 6.70 μM. Compound **21a** displays higher cytotoxic potency than the control drug etoposide (**2**) against four of the five cancer cell lines tested. Some other compounds also exhibit promising antitumor potency against one or more cancer cell lines. Against the HL-60 cancer cell line, compounds **20a**, **24a** and **26b** demonstrate cytotoxicity with an IC_50_ below 10 μM. Most of the other compounds display moderate to weak cytotoxicity against all cancer cells tested.

In our previous study on glucosylated podophyllotoxin derivatives linked via a 4β-triazole ring [24], we have shown that the length of the linker between the glucose moiety and the 1,2,3-triazole residue, the substituents on the glucose residue as well as on the 4*^′^*-position of the E ring can significantly affect the anticancer potency of these compounds. Similar structure-activity relationships are also observed for the series of compounds reported here. The present study also shows that different sugar residues conjugated with 4β-triazolopodophyllotoxin also influence the anticancer activity of these compounds. The most active compound (**21a**) contains a D-galactose residue, and all other compounds containing a D-mannose or D-xylose residue (**24a/b**–**31a/b**) display moderate to weak activity. The majority of the compounds with an α-glycosdic linkage are more active than those with a β-linkage (**20a** *vs*. **20b**, **21a** *vs*. **21b**, **24a** *vs*. **24b**, **28a** *vs*. **28b**).

**Table 2:** *In vitro* anticancer activity (IC_50_, μM) of compounds **20a/b**–**31a/b**.

Compounds	IC_50_ (μM)				
	HL-60	SMMC-7721	A-549	MCF-7	SW480
**20a**	3.02	18.26	18.77	25.00	38.97
**20b**	>40	>40	>40	>40	>40
**21a**	0.49	1.26	1.52	6.70	4.03
**21b**	>40	>40	>40	>40	>40
**22a**	>40	>40	>40	>40	>40
**22b**	>40	>40	>40	>40	>40
**23a**	>40	>40	38.83	36.42	>40
**23b**	>40	>40	>40	>40	>40
**24a**	8.57	14.21	17.86	28.31	>40
**24b**	>40	>40	>40	>40	>40
**25a**	>40	>40	>40	>40	>40
**25b**	>40	>40	>40	>40	>40
**26a**	>40	>40	>40	>40	>40
**26b**	6.85	15.53	18.20	13.61	14.78
**27a**	>40	>40	>40	>40	>40
**27b**	15.27	>40	37.58	28.24	>40
**28a**	14.94	20.18	35.22	31.80	35.35
**28b**	>40	>40	>40	>40	>40
**29a**	>40	>40	>40	>40	>40
**29b**	>40	>40	>40	>40	>40
**30a**	15.01	21.69	18.29	21.56	23.11
**30b**	13.77	16.30	17.75	23.38	39.56
**31a**	>40	>40	>40	>40	>40
**31b**	>40	>40	>40	>40	>40
**Etoposide (2)**	0.31	8.12	11.92	32.82	17.11
**Cisplatin**	1.17	6.43	9.24	15.86	13.42

## 3. Experimental Section

### 3.1. General

Melting points were uncorrected. MS data were obtained in the ESI mode on API Qstar Pulsar instrument (MDS Sciqaszex, Concord, ON, Canada). HRMS data were obtained in the ESI mode on a LCMS-IT-TOF instrument (Shimadzu, Kyoto, Japan). NMR spectra were acquired on Bruker AV-400 or DRX-500 or Bruker AVANCE Ш-600 instruments (Bruker BioSpin GmbH, Rheinstetten, Germany), using tetramethylsilane (TMS) as an internal standard. Column chromatography (CC) was performed on flash silica gel (200–300 mesh; Qingdao Makall Group Co., Ltd; Qingdao; China). All reactions were monitored using thin-layer chromatography (TLC) on silica gel plates.

### 3.2. General Procedure for the Synthesis of Compounds **12a/b**–**17a/b**

D-sugar (5 mmol) was suspended in propargyl alcohol **4**/**5** (25 mmol) and stirred at 65 °C. H_2_SO_4_-silica (25 mg) was added and stirring was continued until all solids had dissolved (~2.5 h). After cooling to room temperature, the reaction mixture was transferred to a short silica gel column (CHCl_3_:CH_3_OH = 15:1→9:1) to afford the desired propargyl glycosides **6**–**11**. Then, to a solution of a propargyl glycosides **6**–**11** (1 mmol) in pyridine (4 mL) at 0 °C butyryl anhydride (4 mL) was added. The reaction mixture was stirred overnight until the starting material disappeared as indicated by TLC. The reaction mixture was diluted with water (20 mL) and extracted with ethyl acetate (3 × 20 mL). The organic layer was washed with 10% aqueous hydrochloric acid (20 mL) and brine (20 mL). The organic layer was dried over magnesium sulfate and evaporated to give a residue, which was chromatographed on silica gel with petroleum ether-acetone = 4:1→2:1 to give the perbutyrylated product **12\a/b***–***17a/b**.

#### 3.2.1. 2-Propyn-1-yl-per-*O*-butyryl-α-d-galactopyranose (**12a**)

Yield: 56%. ^1^H-NMR (CDCl_3_, 400 MHz) δ 5.50 (d, 1H, *J* = 2.7 Hz, C^4^-H), 5.36 (dd, 1H, *J* = 4.0 Hz, 10.0 Hz, C^3^-H), 5.32 (d, 1H, *J* = 4.0 Hz, C^1^-H), 5.15 (dd, 1H, *J* = 4.0 Hz, 10.0 Hz, C^2^-H), 4.39–4.32 (m, 3H), 4.12 (d, 2H, *J* = 7.2 Hz, C*H*_2_-C≡CH), 2.92 (t, 1H, *J* = 2.2 Hz, C≡CH), 2.42 (t, 2H, *J* = 8.0 Hz, COCH_2_), 2.31 (m, 4H, 2 × COCH_2_), 2.20 (t, 2H, *J* = 8.0 Hz, COCH_2_), 1.68–1.58 (m, 8H, 4 × C*H*_2_CH_3_), 1.00–0.92 (m, 12H, 4 × CH_2_C*H*_3_); ^13^C-NMR (CD_3_OD, 100 MHz) δ 174.3 (C=O), 174.2 (C=O), 174.1 (C=O), 173.7 (C=O), 96.1 (C-1), 79.6 (*C*≡CH), 77.0 (C≡*C*H), 69.2, 69.0, 68.7, 68.2, 62.5 (C-6), 56.0 (*C*H_2_-C≡C), 36.8 (*C*H_2_C=O), 36.8 (*C*H_2_C=O), 36.8 (*C*H_2_C=O), 36.7 (*C*H_2_C=O), 19.7 (*C*H_2_CH_3_), 19.6 (*C*H_2_CH_3_), 19.4 (*C*H_2_CH_3_), 19.2 (*C*H_2_CH_3_), 14.3 (CH_2_*C*H_3_), 14.2 (CH_2_*C*H_3_), 14.2 (CH_2_*C*H_3_), 14.2 (CH_2_*C*H_3_); ESIMS: *m/z* 521 [M+Na]^+^, HRESIMS: calcd for C_25_H_38_O_10_Na [M+Na]^+^ 521.2357, found 521.2366.

#### 3.2.2. 2-Propyn-1-yl-per-*O*-butyryl-β-d-galactopyranose (**12b**)

Yield: 33%. ^1^H-NMR (CDCl_3_, 400 MHz) δ 5.42 (d, 1H, *J* = 2.8 Hz, C^4^-H), 5.19 (dd, 1H, *J* = 4.0 Hz, 10.0 Hz, C^3^-H), 5.14 (d, 1H, *J* = 8.0 Hz, C^1^-H), 4.86–4.84 (m, 2H), 4.35 (d, 2H, *J* = 1.9 Hz), 4.14 (s, 2H, C*H*_2_-C≡CH), 2.93 (t, 1H, *J* = 2.2 Hz, C≡CH), 2.42–2.18 (m, 8H, 4 × COCH_2_), 1.71–1.53 (m, 8H, 4 × C*H*_2_CH_3_), 0.99–0.89 (m, 12H, 4 × CH_2_C*H*_3_); ^13^C-NMR (CD_3_OD, 100 MHz) δ 174.5 (C=O), 174.3 (C=O), 173.8 (C=O), 173.7 (C=O), 100.0 (C-1), 79.4 (*C*≡CH), 76.8 (C≡*C*H), 72.2, 72.0, 70.0, 68.6, 62.3 (C-6), 56.8 (*C*H_2_-C≡C), 36.9 (CO*C*H_2_), 36.7 (CO*C*H_2_), 36.7 (CO*C*H_2_), 36.7 (CO*C*H_2_), 19.6 (*C*H_2_CH_3_), 19.5 (*C*H_2_CH_3_), 19.3 (*C*H_2_CH_3_), 19.1 (*C*H_2_CH_3_), 14.0 (CH_2_*C*H_3_), 14.0 (CH_2_*C*H_3_), 14.0 (CH_2_*C*H_3_), 14.0 (CH_2_*C*H_3_); ESIMS: *m/z* 521 [M+Na]^+^, HRESIMS: calcd for C_25_H_38_O_10_Na [M+Na]^+^ 521.2357, found 521.2360.

#### 3.2.3. 2-[2-[2-(2-Propyn-1-yloxy)ethoxy]ethoxy-per-*O*-butyryl-α-d-galactopyranoside (**13a**)

Yield: 57%. ^1^H-NMR (CDCl_3_, 400 MHz) δ 5.45 (d, 1H, *J* = 2.4 Hz, C^4^-H), 5.36 (dd, 1H, *J* = 4.0 Hz, 10.0 Hz, C^3^-H), 5.14–5.10 (m, 2H, C^1^-H, C^2^-H), 4.14 (t, 1H, *J* = 8.0 Hz), 4.18 (d, 2H, *J* = 2.4 Hz, C*H*_2_-C≡CH), 4.09 (dd, 1H, *J* = 6.0 Hz, 10.0 Hz,), 3.84–3.80 (m, 1H), 3.66–3.63 (m, 12H, 3 × OCH_2_CH_2_O), 2.83 (t, 1H, *J* = 2.0 Hz, C≡CH), 2.39 (t, 2H, *J* = 8.0 Hz, COCH_2_), 2.30–2.28 (m, 4H, 2 × COCH_2_), 2.18 (t, 2H, *J* = 8.0 Hz, COCH_2_), 1.69–1.54 (m, 8H, 4 × C*H*_2_CH_3_), 0.98–0.90 (m, 12H, 4 × CH_2_C*H*_3_); ^13^C-NMR (CD_3_OD, 100 MHz) 174.5 (C=O), 174.3 (C=O), 174.3 (C=O), 173.8 (C=O), 97.7 (C^1^-H), 82.7 (*C*≡CH), 76.0 (C≡*C*H), 71.6, 71.6, 71.4, 71.2, 70.1, 69.4, 69.2, 68.9, 68.6, 67.6, 62.6 (C-6), 59.0 (*C*H_2_-C≡C), 37.0 (CO*C*H_2_), 36.8 (CO*C*H_2_), 36.7 (CO*C*H_2_), 36.6 (CO*C*H_2_), 19.6 (*C*H_2_CH_3_), 19.5 (*C*H_2_CH_3_), 19.3 (*C*H_2_CH_3_), 19.2 (*C*H_2_CH_3_), 14.0 (CH_2_*C*H_3_), 14.0 (CH_2_*C*H_3_), 14.0 (CH_2_*C*H_3_), 14.0 (CH_2_*C*H_3_); ESIMS: *m/z* 653 [M+Na]^+^, HRESIMS: calcd for C_31_H_50_O_13_Na [M+Na]^+^ 653.3144, found 653.3149.

#### 3.2.4. 2-[2-[2-(2-Propyn-1-yloxy)ethoxy]ethoxy-per-*O*-butyryl-β-d-galactopyranoside (**13b**)

Yield: 39%. ^1^H-NMR (CDCl_3_, 400 MHz) δ 5.40 (d, 1H, *J* = 2.4 Hz, C^4^-H), 5.13–5.12 (m, 2H, C^3^-H, C^2^-H), 4.73 (d, 1H, *J* = 8.0 Hz, C^1^-H), 4.19 (d, 2H, *J* = 2.0 Hz), 4.12 (s, 2H, C*H*_2_-C≡CH), 3.66–3.60 (m, 12H, 3 × OCH_2_CH_2_O), 2.85 (t, 1H, *J* = 2.0 Hz, C≡CH), 2.41 (t, 2H, *J* = 8.0 Hz, COCH_2_), 2.30–2.29 (m, 4H, 2 × COCH_2_), 2.17 (t, 2H, *J* = 8.0 Hz, COCH_2_), 1.71–1.53 (m, 8H, 4 × C*H*_2_CH_3_), 0.99–0.89 (m, 12H, 4 × CH_2_C*H*_3_); ^13^C-NMR (CD_3_OD, 100 MHz) δ 174.5 (C=O), 174.4 (C=O), 173.8 (C=O), 173.7 (C=O), 102.2 (C-1), 76.0 (C≡*C*H), 72.3, 71.8, 71.6, 71.5, 71.4, 70.2, 70.1, 70.0, 68.6, 62.3 (C-6), 59.0 (*C*H_2_-C≡C), 36.9 (CO*C*H_2_), 36.8 (CO*C*H_2_), 36.7 (CO*C*H_2_), 36.7 (CO*C*H_2_), 19.6 (*C*H_2_CH_3_), 19.5 (*C*H_2_CH_3_), 19.3 (*C*H_2_CH_3_), 19.1 (*C*H_2_CH_3_), 14.0 (CH_2_*C*H_3_), 13.9 (CH_2_*C*H_3_), 13.9 (CH_2_*C*H_3_), 13.9 (CH_2_*C*H_3_); ESIMS: *m/z* 653 [M+Na]^+^, HRESIMS: calcd for C_31_H_50_O_13_Na [M+Na]^+^ 653.3144, found 653.3150.

#### 3.2.5. 2-Propyn-1-yl-per-*O*-butyryl-α-d-mannopyranoside (**14a**)

Yield: 60%. ^1^H-NMR (CDCl_3_, 400 MHz) δ 5.32 (t, 1H, *J* = 10.0 Hz, C^4^-H), 5.23–5.21 (m, 2H, C^3^-H, C^2^-H), 4.98 (s, 1H, C^1^-H), 4.30 (t, 2H, *J* = 2.4 Hz, C*H*_2_-C≡CH), 4.21 (dd, 1H, *J* = 4.0 Hz, 10.0 Hz), 4.12–4.08 (m, 1H), 4.03–4.00 (m, 1H,), 2.92 (t, 1H, *J* = 2.4 Hz, C≡CH), 2.37 (t, 2H, *J* = 8.0 Hz, COCH_2_), 2.30–2.24 (m, 4H, 2 × COCH_2_), 2.15 (t, 2H, *J* = 8.0 Hz, COCH_2_), 1.69–1.50 (m, 8H, 4 × C*H*_2_CH_3_), 0.97–0.86 (m, 12H, 4 × CH_2_C*H*_3_); ^13^C-NMR (CD_3_OD, 100 MHz) δ 174.7 (C=O), 173.8 (C=O), 173.8 (C=O), 173.7 (C=O), 97.5 (C-1), 79.3 (*C*≡CH), 77.0 (C≡*C*H), 70.6, 70.4, 70.3, 66.6, 62.9 (C-6), 55.7 (*C*H_2_-C≡C), 36.9 (CO*C*H_2_), 36.9 (CO*C*H_2_), 36.8 (CO*C*H_2_), 36.7 (CO*C*H_2_), 19.6 (*C*H_2_CH_3_), 19.4 (*C*H_2_CH_3_), 19.3 (*C*H_2_CH_3_), 19.1 (*C*H_2_CH_3_), 14.1 (CH_2_*C*H_3_), 14.0 (CH_2_*C*H_3_), 13.9 (CH_2_*C*H_3_), 13.9 (CH_2_*C*H_3_); ESIMS: *m/z* 521 [M+Na]^+^, HRESIMS: calcd for C_25_H_38_O_10_Na [M+Na]^+^ 521.2357, found 521.2363.

#### 3.2.6. 2-Propyn-1-yl-per-*O*-butyryl-β-d-mannopyranoside (**14b**)

Yield: 34%. ^1^H-NMR (CDCl_3_, 400 MHz) δ 5.44 (d, 1H, *J* = 3.2 Hz, C^2^-H), 5.28 (t, 1H, *J* = 10.0 Hz, C^4^-H), 5.20 (dd, 1H, *J* = 4.0 Hz, 10.0 Hz, C^3^-H), 5.06 (s, 1H, C^1^-H), 4.35 (d, 2H, *J* = 2.4 Hz, C*H*_2_-C≡CH), 4.26 (dd, 1H, *J* = 4.0 Hz. 10.0 Hz), 4.19–4.16 (m, 1H), 3.86–3.82 (m, 1H), 2.94 (t, 1H, *J* = 2.4 Hz, C≡CH), 2.40 (t, 2H, *J* = 8.0 Hz, COCH_2_), 2.34 (t, 2H, *J* = 8.0 Hz, COCH_2_), 2.28 (t, 2H, *J* = 8.0 Hz, COCH_2_), 2.18 (t, 2H, *J* = 8.0 Hz, COCH_2_), 1.72–1.64 (m, 4H, 2 × C*H*_2_CH_3_), 1.62–1.53 (m, 4H, 2 × C*H*_2_CH_3_), 1.00–0.89 (m, 12H, 4 × CH_2_C*H*_3_); ^13^C-NMR (CD_3_OD, 100 MHz) δ 174.8 (C=O), 174.4 (C=O), 173.8 (C=O), 173.7 (C=O), 97.3 (C-1), 79.1 (*C*≡CH), 77.1 (C≡*C*H), 73.5, 82.5, 70.2, 66.8, 62.9 (C-6), 56.7 (*C*H_2_-C≡C), 36.9 (CO*C*H_2_), 36.9 (CO*C*H_2_), 36.8 (CO*C*H_2_), 36.8 (CO*C*H_2_), 19.6 (*C*H_2_CH_3_), 19.4 (*C*H_2_CH_3_), 19.3 (*C*H_2_CH_3_), 19.1 (*C*H_2_CH_3_), 14.0 (CH_2_*C*H_3_), 14.0 (CH_2_*C*H_3_), 14.0 (CH_2_*C*H_3_), 13.9 (CH_2_*C*H_3_); ESIMS: *m/z* 521 [M+Na]^+^, HRESIMS: calcd for C_25_H_38_O_10_Na [M+Na]^+^ 521.2357, found 521.2364.

#### 3.2.7. 2-[2-[2-(2-Propyn-1-yloxy)ethoxy]ethoxy-per-*O*-butyryl-α-d-mannopyranoside (**15a**)

Yield: 62%. ^1^H-NMR (CDCl_3_, 400 MHz) δ 5.34 (t, 1H, *J* = 10.0 Hz, C^4^-H), 5.30 (d, 1H, *J* = 3.2 Hz, C^2^-H), 5.28–5.27 (m, 2H, C^3^-H, C^1^-H), 4.22–4.20 (m, 1H), 4.19 (d, 2H, *J* = 2.4 Hz, C*H*_2_-C≡CH), 4.15–4.12 (m, 1H), 3.88–3.84 (m, 1H), 3.71–3.66 (m, 12H, 3 × OCH_2_CH_2_O), 2.85 (t, 1H, *J* = 2.4 Hz, C≡CH), 2.41 (t, 2H, *J* = 8.0 Hz, COCH_2_), 2.34 (t, 2H, *J* = 8.0 Hz, COCH_2_), 2.32 (t, 2H, *J* = 8.0 Hz, COCH_2_), 2.19 (t, 2H, *J* = 8.0 Hz, COCH_2_), 1.73–1.65 (m, 4H, 2 × C*H*_2_CH_3_), 1.63–1.54 (m, 4H, 2 × C*H*_2_CH_3_), 1.01–0.90 (m, 12H, 4 × CH_2_C*H*_3_); ^13^C-NMR (CD_3_OD, 100 MHz) δ 174.8 (C=O), 173.9 (C=O), 173.8 (C=O), 173.8 (C=O), 99.0 (C-1), 80.7 (*C*≡CH), 76.0 (C≡*C*H), 71.7, 71.6, 71.4, 71.2, 70.8, 70.5, 70.1, 69.9, 68.4, 66.8, 63.1 (C-6), 59.0 (*C*H_2_-C≡C), 36.9 (CO*C*H_2_), 36.9 (CO*C*H_2_), 36.9 (CO*C*H_2_), 36.8 (CO*C*H_2_), 19.6 (*C*H_2_CH_3_), 19.4 (*C*H_2_CH_3_), 19.4 (*C*H_2_CH_3_), 19.2 (*C*H_2_CH_3_), 14.1 (CH_2_*C*H_3_), 14.0 (CH_2_*C*H_3_), 14.0 (CH_2_*C*H_3_), 14.0 (CH_2_*C*H_3_); ESIMS: *m/z* 653 [M+Na]^+^, HRESIMS: calcd for C_31_H_50_O_13_Na [M+Na]^+^ 653.3144, found 653.3149.

#### 3.2.8. 2-[2-[2-(2-Propyn-1-yloxy)ethoxy]ethoxy-per-*O*-butyryl-β-d-mannopyranoside (**15b**)

Yield: 34%. ^1^H-NMR (CDCl_3_, 400 MHz) δ 5.24–5.22 (m, 1H, C^4^-H), 5.13 (dd, 1H, *J* = 4.0 Hz, 10.0 Hz, C^3^-H), 4.86–4.84 (m, 1H, C^2^-H), 4.81 (d, 1H, *J* = 2.0 Hz, C^1^-H), 4.43 (dd, 1H, *J* = 2.0 Hz, 10.0 Hz), 4.29–4.25 (m, 1H), 4.19 (d, 2H, *J* = 2.4 Hz, C*H*_2_-C≡CH), 3.95–3.90 (m, 1H), 3.85–3.82 (m, 1H), 3.69–3.66 (m, 12H, 3 × OCH_2_CH_2_O), 2.85 (t, 1H, *J* = 2.4 Hz, C≡CH), 2.35–2.29 (m, 8H, 4 × COCH_2_), 1.70–1.60 (m, 8H, 4 × C*H*_2_CH_3_), 0.99–0.94 (m, 12H, 4 × CH_2_C*H*_3_); ^13^C-NMR (CD_3_OD, 100 MHz) δ 175.1 (C=O), 174.4 (C=O), 174.4 (C=O), 174.0 (C=O), 99.0 (C-1), 80.7 (*C*≡CH), 76.0 (C≡*C*H), 73.0, 72.1, 71.7, 71.6, 71.4, 71.3, 70.8, 70.1, 68.2, 66.0, 64.2 (C-6), 59.0 (*C*H_2_-C≡C), 37.0 (CO*C*H_2_), 36.9 (CO*C*H_2_), 36.9 (CO*C*H_2_), 36.9 (CO*C*H_2_), 19.6 (*C*H_2_CH_3_), 19.5 (*C*H_2_CH_3_), 19.4 (*C*H_2_CH_3_), 19.2 (*C*H_2_CH_3_), 14.1 (CH_2_*C*H_3_), 14.0 (CH_2_*C*H_3_), 14.0 (CH_2_*C*H_3_), 14.0 (CH_2_*C*H_3_); ESIMS: *m/z* 653 [M+Na]^+^, HRESIMS: calcd for C_31_H_50_O_13_Na [M+Na]^+^ 653.3144, found 653.3144.

#### 3.2.9. 2-Propyn-1-yl-per-*O*-butyryl-α-d-xylopyranoside (**16a**)

Yield: 61%. ^1^H-NMR (CDCl_3_, 400 MHz) δ 5.46 (t, 1H, *J* = 10.0 Hz, C^3^-H), 5.23 (d, 1H, *J* = 4.0 Hz, C^1^-H), 5.04–4.97 (m, 1H, C^2^-H), 4.89–4.85 (m, 1H, C^4^-H), 4.36–4.24 (m, 2H, C*H*_2_-C≡CH), 3.80 (dd, 1H, *J* = 6.0 Hz, 10.0 Hz), 3.63 (t, 1H, *J* = 10.0 Hz), 2.91 (t, 1H, *J* = 2.4 Hz, C≡CH), 2.30–2.25 (m, 6H, 3 × COCH_2_), 1.64–1.57 (m, 6H, 3 × C*H*_2_CH_3_) 0.94–0.92 (m, 9H, 3 × CH_2_C*H*_3_); ^13^C-NMR (CD_3_OD, 100 MHz) δ 174.0 (C=O), 174.0 (C=O), 173.8 (C=O), 95.6 (C-1), 79.5 (*C*≡CH), 76.7 (C≡*C*H), 71.9, 70.5, 70.3, 59.8 (C-6), 55.8 (*C*H_2_-C≡C), 36.9 (CO*C*H_2_), 36.7 (CO*C*H_2_), 36.7 (CO*C*H_2_), 19.4 (*C*H_2_CH_3_), 19.4 (*C*H_2_CH_3_), 19.3 (*C*H_2_CH_3_), 14.0 (CH_2_*C*H_3_), 14.0 (CH_2_*C*H_3_), 14.0 (CH_2_*C*H_3_); ESIMS: *m/z* 421 [M+Na]^+^, HRESIMS: calcd for C_20_H_30_O_8_Na [M+Na]^+^ 421.1833, found 421.1838.

#### 3.2.10. 2-Propyn-1-yl-per-*O*-butyryl-β-d-xylopyranoside (**16b**)

Yield: 29%. ^1^H-NMR (CDCl_3_, 400 MHz) δ 5.26 (t, 1H, *J* = 9.0 Hz, C^3^-H), 4.96–4.88 (m, 2H, C^4^-H, C^2^-H), 4.79 (d, 1H, *J* = 8.0 Hz, C^1^-H), 4.33 (t, 2H, *J* = 1.6 Hz, C*H*_2_-C≡CH), 4.08 (dd, 1H, *J* = 5.0 Hz, 12.0 Hz), 3.47 (dd, 1H, *J* = 10.0 Hz, 12.0 Hz), 2.93 (t, 1H, *J* = 2.4 Hz, C≡CH), 2.29–2.23 (m, 6H, 3 × COCH_2_), 1.65–1.55 (m, 6H, 3 × C*H*_2_CH_3_), 0.94–0.91 (m, 9H, 3 × CH_2_C*H*_3_); ^13^C-NMR (CD_3_OD, 100 MHz) δ 173.9 (C=O), 193.9 (C=O), 173.6 (C=O), 100.1 (C-1), 79.4 (*C*≡CH), 76.7 (C≡*C*H), 73.0, 72.0, 70.2, 63.3 (C-5), 56.7 (*C*H_2_-C≡C), 36.9 (CO*C*H_2_), 36.8 (CO*C*H_2_), 36.7 (CO*C*H_2_), 19.4 (*C*H_2_CH_3_), 19.3 (*C*H_2_CH_3_), 19.3 (*C*H_2_CH_3_), 14.0 (CH_2_*C*H_3_), 14.0 (CH_2_*C*H_3_), 13.9 (CH_2_*C*H_3_); ESIMS: *m/z* 421 [M+Na]^+^, HRESIMS: calcd for C_20_H_30_O_8_Na [M+Na]^+^ 421.1833, found 421.1836.

#### 3.2.11. 2-[2-[2-(2-Propyn-1-yloxy)ethoxy]ethoxy-per-*O*-butyryl-α-d-xylopyranoside (**17a**)

Yield: 62%. ^1^H-NMR (CDCl_3_, 400 MHz) δ 5.48 (t, 1H, *J* = 10.0 Hz, C^3^-H), 5.08 (d, 1H, *J* = 4.0 Hz, C^1^-H), 5.00–4.94 (m, 1H, C^4^-H), 4.83 (dd, 1H, *J* = 4.0 Hz, 10.0 Hz, C^2^-H), 4.19 (d, 2H, *J* = 2.4 Hz, C*H*_2_-C≡CH), 3.85–3.80 (m, 1H), 3.75–3.73 (m, 1H), 3.71–3.65 (m, 12H, 3 × OCH_2_CH_2_O), 2.86 (t, 1H, *J* = 2.0 Hz, C≡CH), 2.32–2.24 (m, 6H, 3 × COCH_2_), 1.64–1.56 (m, 6H, 3 × C*H*_2_CH_3_), 0.95–0.90 (m, 9H, 3 × CH_2_C*H*_3_); ^13^C-NMR (CD_3_OD, 100 MHz) δ 174.1 (C=O), 174.0 (C=O), 173.9 (C=O), 97.3 (C-1), 80.7 (*C*≡CH), 76.0 (C≡*C*H), 72.2, 71.7, 71.6, 71.5, 71.3, 70.7, 70.5, 70.1, 68.6, 59.4 (C-5), 59.1 (*C*H_2_-C≡C), 36.9 (CO*C*H_2_), 36.8 (CO*C*H_2_), 36.7 (CO*C*H_2_), 19.4 (*C*H_2_CH_3_), 19.3 (*C*H_2_CH_3_), 19.3 (*C*H_2_CH_3_), 14.0 (CH_2_*C*H_3_), 13.9 (CH_2_*C*H_3_), 13.9 (CH_2_*C*H_3_); ESIMS: *m/z* 553 [M+Na]^+^, HRESIMS: calcd for C_26_H_42_O_11_Na [M+Na]^+^ 553.2619, found 553.2625.

#### 3.2.12. 2-[2-[2-(2-Propyn-1-yloxy)ethoxy]ethoxy-per-*O*-butyryl-β-d-xylopyranoside (**17b**)

Yield: 28%. ^1^H-NMR (CDCl_3_, 400 MHz) δ 5.23 (t, 1H, *J* = 9.0 Hz, C^3^-H), 4.96–4.86 (m, 2H, C^2^-H, C^4^-H), 4.65 (d, 1H, *J* = 8.0 Hz, C^1^-H), 4.19 (d, 2H, *J* = 2.4 Hz, CH_2_C≡CH), 4.06 (dd, 1H, *J* = 6.0 Hz, 12.0 Hz), 3.91–3.86 (m, 1H), 3.66–3.61 (m, 12H, 3 × OCH_2_CH_2_O), 2.86 (t, 1H, *J* = 2.4 Hz, C≡CH), 2.31–2.22 (m, 6H, 3 × COCH_2_), 1.63–1.54 (m, 6H, 3 × C*H*_2_CH_3_), 0.93–0.90 (m, 9H, 3 × CH_2_C*H*_3_); ^13^C-NMR (CD_3_OD, 100 MHz) δ 173.9 (C=O), 173.9 (C=O), 173.6 (C=O), 102.3 (C-1), 80.7 (*C*≡CH), 76.0 (C≡*C*H), 73.1, 72.4, 71.6, 71.6, 71.4, 71.4, 70.3, 70.1, 69.9, 63.4 (C-5), 59.1 (*C*H_2_-C≡C), 36.9 (CO*C*H_2_), 36.9 (CO*C*H_2_), 36.7 (CO*C*H_2_), 19.4 (*C*H_2_CH_3_), 19.4 (*C*H_2_CH_3_), 19.3 (*C*H_2_CH_3_), 14.0 (CH_2_*C*H_3_), 14.0 (CH_2_*C*H_3_), 13.9 (CH_2_*C*H_3_); ESIMS: *m/z* 553 [M+Na]^+^, HRESIMS: calcd for C_26_H_42_O_11_Na [M+Na]^+^ 553.2619, found 553.2627.

### 3.3. Click Chemistry-General Procedure

To a solution of a terminal-alkyne **12a/b**–**17a/b** (0.1 mmol) and 4β-azidopodophyllotoxin analogues **18** or **19** (0.1 mmol) in *t−*BuOH-H_2_O (1:2, 1.0 mL) at room temperature were added copper (II) sulfate pentahydrate (0.01 mmol) and sodium ascorbate (1.0 M in H_2_O, 3 drops). The reaction mixture was stirred at room temperature for 2 h until the starting material disappeared as indicated by TLC. Then, the mixture was diluted with water (10 mL) and extracted with ethyl acetate (3 × 10 mL), and the combined organic layer was dried over sodium sulfate. The solvent was evaporated and the residue was purified by column chromatography to afford the cycloaddition product **20a/b**–**31a/b** (82%–92%).

#### 3.3.1. 4β-{4^''^-[1^'''^-(2^'''^,3^'''^,4^'''^,6^'''^-Tetra-*O*-butyryl-α-d-galactopyranosyloxy)-1,2,3-triazol-1-yl]}-4-deoxy-podophyllotoxin (**20a**)

White amorphous powder, yield 90% (after chromatography with petroleum ether/acetone, 1:1); mp 87 °C; ${\left[\text{α}\right]}_{D}^{25.7}$: +28.7 (c 0.27, CH_3_OH); ^1^H-NMR (CD_3_OD, 500 MHz) δ 7.87 (s, 1H, C^5''^-H), 6.71 (s, 1H, C^5^-H), 6.61 (s, 1H, C^8^-H), 6.43 (s, 2H, C^2'^, C^6'^-H), 6.26 (d, 1H, *J* = 5.0 Hz, C^4^-H), 5.98 (d, 2H, *J* = 10.0 Hz, OCH_2_O), 5.50 (d, 1H, *J* = 4.0 Hz, C^4'''^-H), 5.36 (dd, 1H, *J* = 4.0 Hz, 10.0 Hz, C^3'''^-H), 5.26 (d, 1H, *J* = 4.0 Hz, C^1'''^-H), 5.00 (dd, 1H, *J* = 4.0 Hz, 10.0 Hz, C^2'''^-H), 4.74–4.72 (m, 2H), 4.31 (d, 1H, *J* = 5.5 Hz, C^1^-H), 4.10–4.05 (m, 1H), 4.01–3.99 (m, 1H), 3.74 (s, 2H), 3.75 (s, 6H, C^3'^, C^5'^-OCH_3_), 3.72 (s, 3H, C^4'^-OCH_3_), 3.39 (dd, 1H, *J* = 4.0 Hz, 10.0 Hz, C^2^-H), 3.15–3.11 (m, 1H, C^3^-H), 2.34 (t, 2H, *J* = 9.0 Hz, COCH_2_), 2.23 (t, 2H, *J* = 9.0 Hz, COCH_2_), 2.16–2.14 (m, 4H, 2 × COCH_2_), 1.69–1.58 (m, 2H, C*H*_2_CH_3_), 1.55–1.48 (m, 6H, 3 × C*H*_2_CH_3_), 0.92–0.82 (m, 12H, 4 × CH_2_C*H*_3_); ^13^C-NMR (CD_3_OD, 125 MHz) δ 174.2 (C-12), 173.0 (C=O), 172.7 (C=O), 172.6 (C=O), 172.3 (C=O), 152.4 (C-3^'^, C-5^'^), 149.0 (C-7), 147.7 (C-6), 143.1 (C-4^''^), 136.7 (C-1^'^), 135.2 (C-9), 133.2 (C-10), 125.4 (C-4^'^), 124.8 (C-5^''^), 109.7 (C-5), 108.3 (C-8), 107.8 (C-2^'^, C-6^'^), 101.8 (OCH_2_O), 95.0 (C-1^'''^), 67.7, 67.6, 67.3 (C-11), 67.2, 66.3, 60.9 (C-6^''^), 59.9 (C-6^'''^), 59.5 (4^'^-OCH_3_), 58.3 (C-2), 55.1 (3^'^, 5^'^-OCH_3_), 43.4 (C-4), 41.0 (C-1), 37.0 (C-3), 35.2 (CO*C*H_2_), 35.2 (CO*C*H_2_), 35.1 (CO*C*H_2_), 35.1 (CO*C*H_2_), 18.0 (*C*H_2_CH_3_), 17.9 (*C*H_2_CH_3_), 17.8 (*C*H_2_CH_3_), 17.6 (*C*H_2_CH_3_), 12.5 (CH_2_*C*H_3_), 12.5 (CH_2_*C*H_3_), 12.4 (CH_2_*C*H_3_), 12.4 (CH_2_*C*H_3_); ESIMS: *m/z* 960 [M+Na]^+^, HRESIMS: calcd for C_47_H_59_N_3_O_17_H [M+H]^+^ 938.3917, found 938.3915.

#### 3.3.2. 4β-{4^''^-[1^'''^-(2^'''^,3^'''^,4^'''^,6^'''^-Tetra-*O*-butyryl-β-d-galactopyranosyloxy)-1,2,3-triazol-1-yl]}-4-deoxy-podophyllotoxin (**20b**)

White amorphous powder, yield 90% (after chromatography with petroleum ether/acetone, 1:1); mp 92 °C; ${\left[\text{α}\right]}_{D}^{25.8}$: −33.2 (c 0.16, CH_3_OH); ^1^H-NMR (CD_3_OD, 400 MHz) δ 7.72 (s,1H, C^5''^-H), 6.67 (s, 1H, C^5^-H), 6.58 (s, 1H, C^8^-H), 6.41 (s, 2H, C^2'^, C^6'^-H), 6.24 (d, 1H, *J* = 4.3 Hz, C^4^-H), 5.94 (d, 2H, *J* = 7.4 Hz, OCH_2_O), 5.42 (d, 1H, *J* = 2.6 Hz, C^4'''^-H), 5.18–5.09 (m, 2H, C^3'''^-H, C^2'''^-H), 4.79 (d, 1H, *J* = 8.0 Hz, C^1'''^-H), 4.78–4.77 (m, 2H), 3.34–3.32 (m, 1H), 4.18–4.11 (m, 2H), 3.79 (s, 2H), 3.72 (s, 6H, C^3'^, C^5'^-OCH_3_), 3.70 (s, 3H, C^4'^-OCH_3_), 3.39 (dd, 1H, *J* = 4.0 Hz, 10.0 Hz, C^2^-H), 3.15–3.11 (m, 1H, C^3^-H), 2.38 (t, 2H, *J* = 8.0 Hz, COCH_2_), 2.27 (t, 2H, *J* = 8.0 Hz, COCH_2_), 2.17–2.15 (m, 4H, 2 × COCH_2_), 1.67–1.47 (m, 8H, 4 × C*H*_2_CH_3_); 0.96–0.82 (m, 12H, 4 × CH_2_C*H*_3_); ^13^C-NMR (CD_3_OD, 100 MHz) δ 175.6 (C-12), 174.4 (C=O), 174.3 (C=O), 173.7 (C=O), 173.7 (C=O), 154.0 (C-3^'^, C-5^'^), 150.6 (C-7), 149.3 (C-6), 145.5 (C-4^''^), 138.6 (C-1^'^), 136.7 (C-9), 134.8 (C-10), 126.9 (C-4^'^), 126.0 (C-5^''^), 111.3 (C-5), 109.9 (C-8), 109.5 (C-2^'^, C-6^'^), 103.3 (OCH_2_O), 101.5 (C-1^'''^), 72.1, 72.0, 70.1, 68.9 (C-11), 68.6, 63.3 (C-6^''^), 62.3 (C-6^'''^), 61.1 (4^'^-OCH_3_), 59.8 (C-2), 56.7 (3^'^, 5^'^-OCH_3_), 44.9 (C-4), 42.5 (C-1), 38.6 (C-3), 36.8 (CO*C*H_2_), 36.7 (CO*C*H_2_), 36.7 (CO*C*H_2_), 36.7 (CO*C*H_2_), 19.6 (*C*H_2_CH_3_), 19.5 (*C*H_2_CH_3_), 19.3 (*C*H_2_CH_3_), 19.1 (*C*H_2_CH_3_), 14.1 (CH_2_*C*H_3_), 14.0 (CH_2_*C*H_3_), 14.0 (CH_2_*C*H_3_), 14.0 (CH_2_*C*H_3_); ESIMS: *m/z* 960 [M+Na]^+^, HRESIMS: calcd for C_47_H_59_N_3_O_17_Na [M+Na]^+^ 938.3917, found 938.3898.

#### 3.3.3. 4β-{4^''^-[1^'''^-(2^'''^,3^'''^,4^'''^,6^'''^-Tetra-*O*-butyryl-α-d-galactopyranosyloxy)-1,2,3-triazol-1-yl]}-4-deoxy-4^'^-demethylpodophyllotoxin (**21a**)

White amorphous powder, yield 89% (after chromatography with petroleum ether/acetone, 1:1); mp 89 °C; ${\left[\text{α}\right]}_{D}^{25.8}$: +22.2 (c 0.22, CH_3_OH); ^1^H-NMR (CD_3_OD, 500 MHz) δ 8.24 (s, 1H, C^5''^-H), 6.61 (s, 3H, C^5^-H, C^2^^'^, C^6^^'^-H), 6.24 (s, 1H, C^8^-H), 5.99–5.91 (m, 3H, C^4^-H, OCH_2_O), 5.52 (d, 1H, *J* = 4.0 Hz, C^4'''^-H), 5.42 (dd, 1H, *J* = 4.0 Hz, 10.0 Hz, C^3^^'''^-H), 5.32 (d. 1H, *J* = 4.0 Hz. C^1^^'''^-H), 5.12 (dd, 1H, *J* = 4.0 Hz, 10.0 Hz, C^2'''^-H), 4.80–4.77 (m, 2H), 4.67 (d, 1H, *J* = 5.0 Hz, C^1^-H), 4.43 (t, 1H, *J* = 8.0 Hz, C^5'''^-H), 4.25 (t, 1H, *J* = 8.0 Hz), 4.21–4.17 (m, 1H), 4.16–4.06 (m, 2H), 3.79 (s, 6H, C^3'^, C^5'^-OCH_3_), 3.57–3.49 (m, 1H, C^3^-H), 3.19 (dd, 1H, *J* = 5.0 Hz, 10.0 Hz, C^2^-H), 2.42 (t, 2H, *J* = 8.0 Hz, COCH_2_), 2.29–2.20 (m, 6H, 3 × COCH_2_), 1.73–1.66 (m, 2H, C*H*_2_CH_3_), 1.61–1.52 (m, 6H, 3 × C*H*_2_CH_3_), 1.00–0.86 (m, 12H, 4 × CH_2_C*H*_3_); ^13^C-NMR (CD_3_OD, 125 MHz) δ 175.9 (C-12), 174.6 (C=O), 174.3 (C=O), 174.2 (C=O), 173.9 (C=O), 149.7 (C-7), 149.1 (C-6), 148.7 (C-3^'^, C-5^'^), 144.9 (C-4^''^), 135.8 (C-1^'^), 134.3 (C-9), 131.7 (C-10), 129.0 (C-4^'^), 125.8 (C-5^''^), 110.0 (C-5), 109.5 (C-2^'^, C-6^'^), 107.3 (C-8), 103.1 (OCH_2_O), 96.9 (C-1^'''^), 71.3 (C-11), 69.3, 69.3, 68.9, 68.0, 64.0 (C-4), 62.5 (C-6^'''^), 61.8 (C-6^''^), 57.0 (3^'^, 5^'^-OCH_3_), 46.6 (C-1), 45.1 (C-2), 40.0 (C-3), 36.8 (CO*C*H_2_), 36.8 (CO*C*H_2_), 36.7 (CO*C*H_2_), 36.7 (CO*C*H_2_), 19.5 (*C*H_2_CH_3_), 19.4 (*C*H_2_CH_3_), 19.3 (*C*H_2_CH_3_), 19.1 (*C*H_2_CH_3_), 14.5 (CH_2_*C*H_3_), 14.0 (CH_2_*C*H_3_), 13.9 (CH_2_*C*H_3_), 13.9 (CH_2_*C*H_3_); ESIMS: *m/z* 946 [M+Na]^+^, HRESIMS: calcd for C_46_H_57_N_3_O_17_Na [M+Na]^+^ 946.3580, found 946.3555.

#### 3.3.4. 4β-{4^''^-[1^'''^-(2^'''^,3^'''^,4^'''^,6^'''^-Tetra-*O*-butyryl-β-d-galactopyranosyloxy)-1,2,3-triazol-1-yl]}-4-deoxy-4^'^-demethylpodophyllotoxin (**21b**)

White amorphous powder, yield 91% (after chromatography with petroleum ether/acetone, 1:1); mp 103–105 °C; ${\left[\text{α}\right]}_{D}^{25.6}$: −45.1 (c 0.27, CH_3_OH); ^1^H-NMR (CD_3_OD, 400 MHz) δ 7.72 (s, 1H, C^5^^''^-H), 6.66 (s, 1H, C^5^-H), 6.61 (s, 1H, C^8^-H), 6.38 (s, 2H, C^2^^'^, C^6^^'^-H), 6.23 (d, 1H, *J* = 3.9 Hz, C^4^-H), 5.95 (d, 2H, *J* = 8.2 Hz, OCH_2_O), 5.42 (d, 1H, *J* = 2.4 Hz, C^4^^'''^-H), 5.14–5.11 (m, 2H, C^3^^'''^-H, C^2^^'''^-H), 4.81 (d, 1H, *J* = 8.0 Hz, C^1^^'''^-H), 4.79–4.75 (m, 3H), 4.36 (m, 1H), 4.17–4.09 (m, 4H), 3.73 (s, 6H, C^3^^'^, C^5^^'^-OCH_3_), 3.35–3.34 (m, 1H, C^2^-H), 3.15–3.11 (m, 1H, C^3^-H), 2.38 (t, 2H, *J* = 7.2 Hz, COCH_2_), 2.27 (t, 2H, *J* = 7.2 Hz, COCH_2_), 2.17 (t, 2H, *J* = 7.2 Hz, COCH_2_), 2.15 (t, 2H, *J* = 7.2 Hz, COCH_2_), 1.68–1.48 (m, 8H, 4 × C*H*_2_CH_3_), 0.97–0.83 (m, 12H, 4 × CH_2_C*H*_3_); ^13^C-NMR (CD_3_OD, 100 MHz) δ 175.8 (C-12), 174.5 (C=O), 174.3 (C=O), 173.8 (C=O), 173.8 (C=O), 150.5 (C-7), 149.2 (C-6), 148.7 (C-3^'^, C-5^'^), 145.4 (C-4^''^), 136.1 (C-1^'^), 135.1 (C-9), 131.3 (C-10), 126.9 (C-4^'^), 126.0 (C-5^''^), 111.3 (C-5), 109.8 (C-8), 109.4 (C-2^'^, C-6^'^), 103.3 (OCH_2_O), 101.5 (C-1^'''^), 72.1, 72.0, 70.1, 62.2, 68.5 (C-11), 63.2 (C-6^''^), 62.2 (C-6^'''^), 59.9 (C-2), 56.8 (3^'^, 5^'^-OCH_3_), 44.8 (C-4), 42.7 (C-1), 38.5 (C-3), 36.8 (CO*C*H_2_), 36.7 (CO*C*H_2_), 36.7 (CO*C*H_2_), 36.6 (CO*C*H_2_), 19.6 (*C*H_2_CH_3_), 19.4 (*C*H_2_CH_3_), 19.3 (*C*H_2_CH_3_), 19.1 (*C*H_2_CH_3_), 14.0 (CH_2_*C*H_3_), 13.9 (CH_2_*C*H_3_), 13.9 (CH_2_*C*H_3_), 13.9 (CH_2_*C*H_3_); ESIMS: *m/z* 946 [M+Na]^+^, HRESIMS: calcd for C_46_H_57_N_3_O_17_H [M+H]^+^ 924.3761, found 924.3745.

#### 3.3.5. 4β-{4^''^-[1^'''^-(2^'''^,3^'''^,4^'''^,6^'''^-Tetra-*O*-butyryl-α-d-galactopyranosyloxy)-3,6,9-trioxadec-10-yl]-1,2,3-triazol-1-yl}-4-deoxypodophyllotoxin (**22a**)

White amorphous powder, yield 82% (after chromatography with petroleum ether/acetone, 1:1); mp 82 °C; ${\left[\text{α}\right]}_{D}^{25.7}$: −26.2 (c 0.18, CH_3_OH); ^1^H-NMR (CD_3_OD, 400 MHz) δ 7.81 (s, 1H, C^5^^''^-H), 6.70 (s, 1H, C^5^-H), 6.63 (s, 1H, C^8^-H), 6.42 (s, 2H, C^2^^'^, C^6^^'^-H), 6.27 (d, 1H, *J* = 4.8 Hz, C^4^-H), 5.98 (d, 2H, *J* = 8.4 Hz, OCH_2_O), 5.41 (d, 1H, *J* = 1.2 Hz, C^4^^'''^-H), 5.16–5.10 (m, 3H, C^1^^'''^-H, C^3^^'''^-H, C^2^^'''^-H), 4.81 (d, 1H, *J* = 5.2 Hz, C^1^-H), 4.74–4.72 (m, 1H), 4.65–4.63 (m, 2H), 4.41–4.36 (m, 1H), 4.13–4.12 (m, 2H), 3.92–3.86 (m, 1H), 3.81–3.80 (m, 1H), 3.74 (s, 6H, C^3^^'^, C^5^^'^-OCH_3_), 3.72 (s, 3H, C^4^^'^-OCH_3_), 3.66–3.58 (m, 12H, 3 × OCH_2_CH_2_O), 3.43 (dd, 1H, *J* = 1.2 Hz, 10.0 Hz, C^2^-H), 3.19–3.14 (m, 1H, C^3^-H), 3.36 (t, 2H, *J* = 8.0 Hz, COCH_2_), 2.28–2.26 (m, 4H, 2 × COCH_2_), 1.64–1.52 (m, 8H, 4 × C*H*_2_CH_3_), 0.96–0.89 (m, 12H, 4 × CH_2_C*H*_3_); ^13^C-NMR (CD_3_OD, 100 MHz) δ 175.8 (C-12), 174.5 (C=O), 174.3 (C=O), 173.8 (C=O), 173.8 (C=O), 154.0 (C-3^'^, C-5^'^), 150.6 (C-7), 149.3 (C-6), 146.1 (C-4^''^), 138.3 (C-1^'^), 136.8 (C-9), 134.8 (C-10), 127.0 (C-4^'^), 125.9 (C-5^''^), 111.2 (C-5), 109.9 (C-8), 109.4 (C-2^'^, C-6^'^), 103.3 (OCH_2_O), 102.3 (C-1^'''^), 72.3, 71.8, 71.6, 71.5, 71.4, 70.9, 70.2, 70.1, 68.9 (C-11), 68.6, 65.0 (C-6^''^), 63.3 (C-6^'''^), 61.1 (4^'^-OCH_3_), 59.8 (C-2), 56.6 (3^'^, 5^'^-OCH_3_), 44.9 (C-4), 42.5 (C-1), 38.6 (C-3), 36.9 (CO*C*H_2_), 36.7 (CO*C*H_2_), 36.7 (CO*C*H_2_), 36.7 (CO*C*H_2_), 19.6 (*C*H_2_CH_3_), 19.5 (*C*H_2_CH_3_), 19.3 (*C*H_2_CH_3_), 19.1 (*C*H_2_CH_3_), 14.0 (CH_2_*C*H_3_), 13.9 (CH_2_*C*H_3_), 13.9 (CH_2_*C*H_3_), 13.9 (CH_2_*C*H_3_); ESIMS: *m/z* 1092 [M+Na]^+^, HRESIMS: calcd for C_53_H_71_N_3_O_20_Na [M+Na]^+^ 1092.4523, found 1092.4484.

#### 3.3.6. 4β-{4^''^-[1^'''^-(2^'''^,3^'''^,4^'''^,6^'''^-Tetra-*O*-butyryl-β-d-galactopyranosyloxy)-3,6,9-trioxadec-10-yl]-1,2,3-triazol-1-yl}-4-deoxypodophyllotoxin (**22b**)

White amorphous powder, yield 88% (after chromatography with petroleum ether/acetone, 1:1); mp 75 °C; ${\left[\text{α}\right]}_{D}^{25.6}$: −25.5 (c 0.14, CH_3_OH); ^1^H-NMR (CD_3_OD, 400 MHz) δ 7.81 (s,1H, C^5^^''^- H), 6.70 (s, 1H, C^5^-H), 6.62 (s, 1H, C^8^-H), 6.42 (s, 2H, C^2^^'^, C^6^^'^-H), 6.26 (d, 1H, *J* = 4.8 Hz, C^4^-H), 5.96 (d, 2H, *J* = 9.2 Hz, OCH_2_O), 5.41 (d, 1H, *J* = 2.8 Hz, C^4^^'''^-H), 5.16–5.12 (m, 2H, C^3^^'''^-H, C^2^^'''^-H), 4.80 (d, 1H, *J* = 5.2 Hz, C^1^-H), 4.74 (d, 1H, *J* = 7.2 Hz, C^1^^'''^-H), 4.62 (s, 2H), 4.40–4.34 (m, 1H), 4.13-4.12 (m, 2H), 3.91–3.86 (m, 1H), 3.74 (s, 6H, C^3^^'^, C^5^^'^-OCH_3_), 3.72 (s, 3H, C^4^^'^-OCH_3_), 3.66–3.57 (m, 12H, 3 × OCH_2_CH_2_O), 3.44 (dd, 1H, *J* = 5.2 Hz, 10.8 Hz, C^2^-H), 3.18–3.13 (m, 1H, C^3^-H), 3.35 (t, 2H, *J* = 8.0 Hz, COCH_2_), 2.29–2.27 (m, 4H, 2 × COCH_2_), 2.18 (t, 2H, *J* = 7.6 Hz, COCH_2_), 1.67–1.52 (m, 8H, 4 × C*H*_2_CH_3_), 0.95–0.89 (m, 12H, 4 × CH_2_C*H*_3_); ^13^C-NMR (CD_3_OD, 100 MHz) δ 175.7 (C-12), 174.5 (C=O), 174.3 (C=O), 173.8 (C=O), 173.8 (C=O), 154.0 (C-3^'^, C-5^'^), 150.5 (C-7), 149.3 (C-6), 146.1 (C-4^''^), 138.3 (C-1^'^), 136.8 (C-9), 134.8 (C-10), 127.0 (C-4^'^), 125.9 (C-5^''^), 111.2 (C-5), 109.9 (C-8), 109.5 (C-2^'^, C-6^'^), 103.3 (OCH_2_O), 102.3 (C-1^'''^), 72.3, 71.8, 71.6, 71.5, 71.4, 70.9, 70.2, 70.1, 68.9 (C-11), 68.7, 65.0 (C-6^''^), 62.4 (C-6^'''^), 61.1 (4^'^-OCH_3_), 59.8 (C-2), 56.7 (3^'^, 5^'^-OCH_3_), 44.8 (C-4), 42.5 (C-1), 38.6 (C-3), 36.9 (CO*C*H_2_), 36.7 (CO*C*H_2_), 36.7 (CO*C*H_2_), 36.7 (CO*C*H_2_), 19.6 (*C*H_2_CH_3_), 19.5 (*C*H_2_CH_3_), 19.3 (*C*H_2_CH_3_), 19.1 (*C*H_2_CH_3_), 14.0 (CH_2_*C*H_3_), 14.0 (CH_2_*C*H_3_), 13.9 (CH_2_*C*H_3_), 13.9 (CH_2_*C*H_3_); ESIMS: *m/z* 1078 [M+Na]^+^, HRESIMS: calcd for C_52_H_69_N_3_O_20_Na [M+Na]^+^ 1078.4367, found 1078.4345.

#### 3.3.7. 4β-{4^''^-[1^'''^-(2^'''^,3^'''^,4^'''^,6^'''^-Tetra-*O*-butyryl-α-d-galactopyranosyloxy)-3,6,9-trioxadec-10-yl]-1,2,3-triazol-1-yl}-4-deoxy-4^'^-demethylpodophyllotoxin (**23a**)

White amorphous powder, yield 87% (after chromatography with petroleum ether/acetone, 1:1); mp 84–85 °C; ${\left[\text{α}\right]}_{D}^{25.9}$: +6.7 (c 0.23, CH_3_OH); ^1^H-NMR (CD_3_OD, 400 MHz) δ 7.79 (s,1H, C^5^^''^-H), 6.69 (s, 1H, C^5^-H), 6.65 (s, 1H, C^8^-H), 6.38 (s, 2H, C^2^^'^, C^6^^'^-H), 6.26 (d, 1H, *J* = 4.8 Hz, C^4^-H), 5.98 (d, 2H, *J* = 5.6 Hz, OCH_2_O), 5.47 (d, 1H, *J* = 2.4 Hz, C^4^^'''^-H), 5.37 (dd, 1H, *J* = 3.2 Hz, 10.8 Hz, C^2^^'''^-H), 5.15–5.08 (m, 2H, C^1^^'''^-H, C^3^^'''^-H), 4.77 (d, 1H, *J* = 4.4 Hz, C^1^-H), 4.63 (s, 2H), 4.44–4.37 (m, 2H), 4.12–4.06 (m, 1H), 3.84–3.80 (m, 1H), 3.74 (s, 6H, C^3^^'^, C^5^^'^-OCH_3_), 3.67–3.60 (m, 12H, 3 × OCH_2_CH_2_CO), 3.40 (dd, 1H, *J* = 4.4 Hz, 10.8 Hz, C^2^-H), 3.19–3.13 (m, 1H, C^3^-H), 2.39 (t, 2H, *J* = 8.0 Hz, COCH_2_), 2.29–2.27 (m, 4H, 2 × COCH_2_), 2.19 (t, 2H, *J* = 8.0 Hz, COCH_2_), 1.69–1.53 (m, 8H, 2 × C*H*_2_CH_3_), 0.98–0.90 (m, 12H, 4 × CH_2_C*H*_3_); ^13^C-NMR (CD_3_OD, 100 MHz) δ 174.4 (C-12), 173.0 (C=O), 172.8 (C=O), 172.8 (C=O), 172.4 (C=O), 149.0 (C-7), 147.7 (C-6), 147.2 (C-3^'^, C-5^'^), 144.6 (C-4^''^), 134.5 (C-1^'^), 133.6 (C-9), 129.8 (C-10), 125.3 (C-4^'^), 124.2 (C-5^''^), 109.7 (C-5), 108.2 (C-8), 107.8 (C-2^'^, C-6^'^), 101.7 (OCH_2_O), 96.1 (C-1^'''^), 70.0, 69.6, 69.6, 69.4, 67.8, 67.7, 67.3 (C-11), 67.0, 63.5 (C-6^''^), 60.0 (C-6^'''^), 58.3 (C-2), 55.2 (3^'^, 5^'^-OCH_3_), 43.2 (C-4), 41.1 (C-1), 37.0 (C-3), 35.2 (CO*C*H_2_), 35.1 (CO*C*H_2_), 35.1 (CO*C*H_2_), 35.1 (CO*C*H_2_), 18.0 (*C*H_2_CH_3_), 17.9 (*C*H_2_CH_3_), 17.7 (*C*H_2_CH_3_), 17.6 (*C*H_2_CH_3_), 12.4 (CH_2_*C*H_3_), 12.4 (CH_2_*C*H_3_), 12.4 (CH_2_*C*H_3_), 12.4 (CH_2_*C*H_3_); ESIMS: *m/z* 1078 [M+Na]^+^, HRESIMS: calcd for C_52_H_69_N_3_O_20_Na [M+Na]^+^ 1078.4367, found 1078.4345.

#### 3.3.8. 4β-{4^''^-[1^'''^-(2^'''^,3^'''^,4^'''^,6^'''^-Tetra-*O*-butyryl-β-d-galactopyranosyloxy)-3,6,9-trioxadec-10-yl]-1,2,3-triazol-1-yl}-4-deoxy-4^'^-demethylpodophyllotoxin (**23b**)

White amorphous powder, yield 85% (after chromatography with petroleum ether/acetone, 1:1); mp 77°C; ${\left[\text{α}\right]}_{D}^{25.6}$: −21.5 (c 0.29, CH_3_OH); ^1^H-NMR (CD_3_OD, 400 MHz) δ 7.81 (s, 1H, C^5^^''^-H), 6.69 (s, 1H, C^5^-H), 6.63 (s, 1H, C^8^-H), 6.39 (s, 2H, C^2^^'^, C^6^^'^-H), 6.25 (d, 1H, *J* = 4.8 Hz, C^4^-H), 5.96 (d, 2H, *J* = 8.8 Hz, OCH_2_O), 5.41 (d, 1H, *J* = 2.8 Hz, C^4^^'''^-H), 5.16–5.10 (m, 2H, C^3^^'''^-H, C^2^^'''^-H), 4.77 (d, 1H, *J* = 4.8 Hz, C^1^-H), 4.73 (d, 1H, *J* = 7.2 Hz, C^1^^'''^-H), 4.62 (s, 2H), 4.39–4.36 (m, 1H), 4.13 (s, 2H), 3.91–3.86 (m, 1H), 3.74 (s, 6H, C^3^^'^, C^5^^'^-OCH_3_), 3.66–3.57 (m, 12H, 3 × OCH_2_CH_2_CO), 3.40 (dd, 1H, *J* = 4.8Hz, 10.8 Hz, C^2^-H), 3.17–3.13 (m, 1H, C^3^-H), 2.36 (t, 2H, *J* = 7.6 Hz, COCH_2_), 2.29–2.27 (m, 4H, 2 × COCH_2_), 2.18 (t, 2H, *J* = 7.6 Hz, COCH_2_), 1.67–1.52 (m, 8H, 4 × C*H*_2_CH_3_) 0.95–0.89 (m, 12H, 4 × CH_2_C*H*_3_); ^13^C-NMR (CD_3_OD, 100 MHz) δ 174.8 (C-12), 173.4 (C=O), 174.2 (C=O), 172.7 (C=O), 172.7 (C=O), 149.4 (C-7), 148.1 (C-6), 147.6 (C-3^'^, C-5^'^), 145.0 (C-4^''^), 134.9 (C-1^'^), 134.0 (C-9), 130.2 (C-10), 125.8 (C-4^'^), 124.8 (C-5^''^), 110.2 (C-5), 108.7 (C-8), 108.3 (C-2^'^, C-6^'^), 102.1 (OCH_2_O), 101.1 (C-1^'''^), 72.3, 71.8, 71.6, 71.5, 71.4, 70.9, 70.2, 70.1, 68.9 (C-11), 68.6, 65.0 (C-6^''^), 62.4 (C-6^'''^), 59.9 (C-2), 55.7 (3^'^, 5^'^-OCH_3_), 43.6 (C-4), 41.5 (C-1), 37.4 (C-3), 35.8 (CO*C*H_2_), 35.6 (CO*C*H_2_), 35.6 (CO*C*H_2_), 35.5 (CO*C*H_2_), 18.4 (*C*H_2_CH_3_), 18.4 (*C*H_2_CH_3_), 18.2 (*C*H_2_CH_3_), 18.0 (*C*H_2_CH_3_), 12.9 (CH_2_*C*H_3_), 12.8 (CH_2_*C*H_3_), 12.8 (CH_2_*C*H_3_), 12.8 (CH_2_*C*H_3_); ESIMS: *m/z* 1078 [M+Na]^+^, HRESIMS: calcd for C_52_H_69_N_3_O_20_H [M+H]^+^ 1056.4547, found 1056.4528.

#### 3.3.9. 4β-{4^''^-[1^'''^-(2^'''^,3^'''^,4^'''^,6^'''^-Tetra-*O*-butyryl-α-d-mannopyranosyloxy)-1,2,3-triazol-1-yl]}-4-deoxy-podophyllotoxin (**24a**)

White amorphous powder, yield 90% (after chromatography with petroleum ether/acetone, 1:1); mp 80 °C; ${\left[\text{α}\right]}_{D}^{26.8}$: −3.8 (c 0.27, CH_3_OH); ^1^H-NMR (CD_3_OD, 400 MHz) δ 7.85 (s, 1H, C^5^^''^-H), 6.68 (s, 1H, C^5^-H), 6.57 (s, 1H, C^8^-H), 6.40 (s, 2H, C^2^^'^, C^6^^'^-H), 6.24 (d, 1H, *J* = 4.4 Hz, C^4^-H), 5.93 (d, 2H, *J* = 9.2 Hz, OCH_2_O), 5.35 (t, 1H, *J* = 10.0 Hz, C^4^^'''^-H), 5.25 (dd, 1H, *J* = 2.8 Hz, 10.0 Hz, C^3^^'''^-H), 5.19–5.18 (m, 1H, C^2^^'''^-H), 4.93 (d, 1H, *J* = 2.8 Hz, C^1^^'''^-H), 4.78–4.76 (m, 2H), 4.68 (s, 1H, C^1^-H), 4.35 (t, 1H, *J* = 6.8 Hz), 4.22–4.20 (m, 1H), 4.10–4.06 (m, 2H), 3.73 (s, 6H, C^3^^'^, C^5^^'^-OCH_3_), 3.71 (s, 3H, C^4^^'^-OCH_3_), 3.30 (dd, 1H, *J* = 4.8 Hz, 10.4 Hz, C^2^-H), 3.20–3.15 (m, 1H, C^3^-H), 3.38–3.34 (m, 8H, 4 × COCH_2_), 1.69–1.62 (m, 4H, 2 × C*H*_2_CH_3_), 1.60–1.51 (m, 4H, 2 × C*H*_2_CH_3_), 0.97–0.87 (m, 12H, 4 × CH_2_C*H*_3_); ^13^C-NMR (CD_3_OD, 100 MHz) δ 175.7 (C-12), 174.7 (C=O), 174.0 (C=O), 173.9 (C=O), 173.7 (C=O), 154.0 (C-3^'^, C-5^'^), 150.6 (C-7), 149.3 (C-6), 144.9 (C-4^''^), 138.3 (C-1^'^), 136.8 (C-9), 134.8 (C-10), 126.9 (C-4^'^), 126.2 (C-5^''^), 111.2 (C-5), 109.9 (C-8), 109.5 (C-2^'^, C-6^'^), 103.3 (OCH_2_O), 98.2 (C-1^'''^), 70.6, 70.6, 70.2, 68.9 (C-11), 66.6, 62.9 (C-6^''^), 61.7 (C-6^'''^), 61.0 (4^'^-OCH_3_), 59.9 (C-2), 56.6 (3^'^, 5^'^-OCH_3_), 44.9 (C-4), 42.50 (C-1), 38.6 (C-3), 36.8 (CO*C*H_2_), 36.8 (CO*C*H_2_), 36.8 (CO*C*H_2_), 36.7(CO*C*H_2_), 19.6 (*C*H_2_CH_3_), 19.4 (*C*H_2_CH_3_), 19.3 (*C*H_2_CH_3_), 19.2 (*C*H_2_CH_3_), 14.1 (CH_2_*C*H_3_), 14.0 (CH_2_*C*H_3_), 14.0 (CH_2_*C*H_3_), 14.0 (CH_2_*C*H_3_); ESIMS: *m/z* 960 [M+Na]^+^, HRESIMS: calcd for C_47_H_59_N_3_O_17_H [M+H]^+^ 938.3917, found 938.3906.

#### 3.3.10. 4β-{4^''^-[1^'''^-(2^'''^,3^'''^,4^'''^,6^'''^-Tetra-*O*-butyryl-β-d-mannopyranosyloxy)-1,2,3-triazol-1-yl]}-4-deoxy-podophyllotoxin (**24b**)

White amorphous powder, yield 86% (after chromatography with petroleum ether/acetone, 1:1); mp 92–93 °C; ${\left[\text{α}\right]}_{D}^{26.8}$: −52.1 (c 0.17, CH_3_OH); ^1^H-NMR (CD_3_OD, 400 MHz) δ 7.74 (s, 1H, C^5^^''^-H), 6.66 (s, 1H, C^5^-H), 6.59 (s, 1H, C^8^-H), 6.40 (s, 2H, C^2^^'^, C^6^^'^-H), 6.22 (d, 1H, *J* = 4.4 Hz, C^4^-H), 5.94 (d, 2H, *J* = 10.0 Hz, OCH_2_O), 5.42 (d, 1H, *J* = 2.8 Hz, C^2^^'''^-H), 5.26 (t, 1H, *J* = 10.0 Hz, C^4^^'''^-H), 5.16 (dd, 1H, *J* = 2.8 Hz, 10.0 Hz, C^3^^'''^-H), 4.99 (s, 1H, C^1^^'''^-H), 4.84 (s, 1H, C^1^-H), 4.77–4.72 (m, 2H), 4.36–4.32 (m, 1H), 4.25 (dd, 1H, *J* = 4.4 Hz, 10.4 Hz), 4.17–4.12 (m, 1H,), 3.85–3.82 (m, 1H), 3.72 (s, 6H, C^3^^'^, C^5^^'^-OCH_3_), 3.71 (s, 3H, C^4^^'^-OCH_3_), 3.39 (dd, 1H, *J* = 4.8 Hz, 10.0 Hz, C^2^-H), 3.17–3.13 (m, 1H, C^3^-H), 2.34–2.25 (m, 8H, 4 × COCH_2_), 1.66–1.51 (m, 8H, 4 × C*H*_2_CH_3_) 0.94–0.88 (t, 12H, 4 × CH_2_C*H*_3_); ^13^C-NMR (CD_3_OD, 100 MHz) δ 175.7 (C-12), 174.7 (C=O), 174.3 (C=O), 173.8 (C=O), 173.7 (C=O), 154.0 (C-3^'^, C-5^'^), 150.5 (C-7), 149.3 (C-6), 145.3 (C-4^''^), 138.3 (C-1^'^), 136.7 (C-9), 134.8 (C-10), 126.9 (C-5^''^), 126.2 (C-4^'^), 111.2 (C-5), 109.4 (C-8), 109.9 (C-2^'^, C-6^'^), 103.3 (OCH_2_O), 99.3 (C-1^'''^), 73.5, 72.5, 70.3, 69.3 (C-11), 66.8, 63.5 (C-6^''^), 62.9 (C-6^'''^), 61.1 (4^'^-OCH_3_), 59.9 (C-2), 56.6 (3^'^, 5^'^-OCH_3_), 44.9 (C-4), 42.5 (C-1), 38.9 (CO*C*H_2_), 36.9 (CO*C*H_2_), 36.8 (CO*C*H_2_), 36.8 (CO*C*H_2_), 19.7 (*C*H_2_CH_3_), 19.4 (*C*H_2_CH_3_), 19.2 (*C*H_2_CH_3_), 19.2 (*C*H_2_CH_3_), 14.1 (CH_2_*C*H_3_), 14.0 (CH_2_*C*H_3_), 14.0 (CH_2_*C*H_3_), 14.0 (CH_2_*C*H_3_); ESIMS: *m/z* 960 [M+Na]^+^, HRESIMS: calcd for C_47_H_59_N_3_O_17_H [M+H]^+^ 938.3917, found 938.3902.

#### 3.3.11. 4β-{4^''^-[1^'''^-(2^'''^,3^'''^,4^'''^,6^'''^-Tetra-*O*-butyryl-α-d-mannopyranosyloxy)-1,2,3-triazol-1-yl]}-4-deoxy-4^'^-demethylpodophyllotoxin (**25a**)

White amorphous powder, yield 92% (after chromatography with CHCl_3_/CH_3_OH, 9:1); mp 94–96 °C; ${\left[\text{α}\right]}_{D}^{26.7}$: −46.3 (c 0.17, Pyridine); ^1^H-NMR (C_5_D_5_N, 400 MHz) δ 8.32 (s,1H, C^5^^''^-H), 6.82 (s, 1H, C^5^-H), 6.83 (s, 1H, C^8^-H), 6.78 (s, 2H, C^2^^'^, C^6^^'^-H), 6.55 (d, 1H, *J* = 4.8 Hz, C^4^-H), 5.97 (d, 2H, *J* = 4.4 Hz, OCH_2_O), 5.83 (t, 1H, *J* = 10.0 Hz, C^4^^'''^-H), 5.72 (dd, 1H, *J* = 3.2 Hz, 10.0 Hz, C^3^^'''^-H), 5.68–5.67 (m, 1H, C^2^^'''^-H), 5.38 (s, 1H, C^1^^'''^-H), 5.18–5.15 (m, 2H), 5.02 (s, 1H, C^1^-H), 4.97 (t, 1H, *J* = 5.2 Hz), 4.55 (dd, 1H, *J* = 4.8 Hz, 10.0 Hz), 4.48–4.45 (m, 2H), 3.77 (dd, 1H, *J* = 5.2 Hz, 10.8 Hz, C^2^-H), 3.72 (s, 6H, C^3^^'^, C^5^^'^-OCH_3_), 3.50–3.45 (m, 1H, C^3^-H), 2.42–2.38 (m, 4H, 2 × COCH_2_), 2.31–2.26 (m, 6H, 3 × COCH_2_), 1.70–1.56 (m, 8H, 4 × C*H*_2_CH_3_), 0.87–0.80 (m, 12H, 4 × CH_2_C*H*_3_); ^13^C-NMR (C_5_D_5_N, 100 MHz) δ 174.1 (C-12), 173.2 (C=O), 172.7 (C=O), 172.5 (C=O), 172.5 (C=O), 149.5 (C-7), 148.7 (C-3^'^, C-5^'^), 148.2 (C-6), 144.2 (C-4^''^), 137.3 (C-1^'^), 134.6 (C-9), 130.1 (C-10), 126.3 (C-5^''^), 125.2 (C-4^'^), 110.8 (C-5), 109.7 (C-2^'^, C-6^'^), 109.3 (C-8), 102.4 (OCH_2_O), 97.4 (C-1^'''^), 69.9, 69.8, 69.6, 67.9 (C-11), 66.1, 62.3 (C-6^''^), 61.4 (C-6^'''^), 59.0 (C-2), 56.5 (3^'^, 5^'^-OCH_3_), 44.1 (C-4), 42.0 (C-1), 38.0 (C-3), 36.2 (CO*C*H_2_), 36.0 (CO*C*H_2_), 36.0 (CO*C*H_2_), 36.0 (CO*C*H_2_), 18.7 (*C*H_2_CH_3_), 18.7 (*C*H_2_CH_3_), 18.7 (*C*H_2_CH_3_), 18.5 (*C*H_2_CH_3_), 13.8 (CH_2_*C*H_3_), 13.7 (CH_2_*C*H_3_), 13.7 (CH_2_*C*H_3_), 13.6 (CH_2_*C*H_3_); ESIMS: *m/z* 946 [M+Na]^+^, HRESIMS: calcd for C_46_H_57_N_3_O_17_H [M+H]^+^ 924.3761, found 924.3752.

#### 3.3.12. 4β-{4^''^-[1^'''^-(2^'''^,3^'''^,4^'''^,6^'''^-Tetra-*O*-butyryl-β-d-mannopyranosyloxy)-1,2,3-triazol-1-yl]}-4-deoxy-4^'^-demethylpodophyllotoxin (**25b**)

White amorphous powder, yield 87% (after chromatography with petroleum ether/acetone, 1:1); mp 99–100 °C; ${\left[\text{α}\right]}_{D}^{26.8}$: −63.6 (c 0.13, CH_3_OH); ^1^H-NMR (CD_3_OD, 400 MHz) δ 7.73 (s, 1H, C^5^^''^-H), 6.66 (s, 1H, C^5^-H), 6.61 (s, 1H, C^8^-H), 6.38 (s, 2H, C^2^^'^, C^6^^'^-H), 6.22 (d, 1H, *J* = 4.4 Hz, C^4^-H), 5.95 (d, 2H, *J* = 9.2 Hz, OCH_2_O), 5.42 (d, 1H, *J* = 2.8 Hz, C^2^^'''^-H), 5.26 (t, 1H, *J* = 10.0 Hz, C^4^^'''^-H), 5.15 (dd, 1H, *J* = 2.8 Hz, 10.0 Hz, C^3^^'''^-H), 4.99 (s, 1H, C^1^^'''^-H), 4.86 (d, 1H, *J* = 4.4 Hz, C^1^-H), 4.76–4.73 (m, 2H), 4.37–4.34 (m, 1H), 4.26 (dd, 1H, *J* = 4.0 Hz, 10.0 Hz), 4.14 (dd, 1H, *J* = 2.0 Hz, 10.0 Hz), 3.86–3.81 (m, 1H), 3.73 (s, 6H, C^3^^'^, C^5^^'^-OCH_3_), 3.34–3.33 (m, 1H, C^2^-H), 3.18–3.13 (m, 1H, C^3^-H), 2.35–2.26 (m, 8H, 4 × CO*C*H_2_), 1.67–1.52 (m, 8H, 4 × C*H*_2_CH_3_), 0.96–0.88 (m, 12H, 4 × CH_2_C*H*_3_); ^13^C-NMR (CD_3_OD, 100 MHz) δ 175.9 (C-12), 174.7 (C=O), 174.4 (C=O), 173.4 (C=O), 173.7 (C=O), 150.5 (C-7), 149.2 (C-6), 148.7 (C-3^'^, C-5^'^), 145.3 (C-4^''^), 136.0 (C-1^'^), 135.1 (C-9), 131.3 (C-10), 126.8 (C-4^'^), 126.2 (C-5^''^), 111.3 (C-5), 109.8 (C-8), 109.3 (C-2^'^, C-6^'^), 103.3 (OCH_2_O), 99.3 (C-1^'''^), 73.5, 72.4, 70.3, 66.8, 68.9 (C-11), 63.5 (C-6^''^), 62.9 (C-6^'''^), 60.0 (C-2), 56.8 (3^'^, 5^'^-OCH_3_), 44.89 (C-4), 42.7 (C-1), 38.5 (C-3), 36.9 (CO*C*H_2_), 36.8 (CO*C*H_2_), 36.8 (CO*C*H_2_), 36.7 (CO*C*H_2_), 19.7 (*C*H_2_CH_3_), 19.4 (*C*H_2_CH_3_), 19.3 (*C*H_2_CH_3_), 19.2 (*C*H_2_CH_3_), 14.1 (CH_2_*C*H_3_), 14.0 (CH_2_*C*H_3_), 14.0 (CH_2_*C*H_3_), 13.9 (CH_2_*C*H_3_); ESIMS: *m/z* 946 [M+Na]^+^, HRESIMS: calcd for C_46_H_57_N_3_O_17_H [M+H]^+^ 924.3761, found 924.3753.

#### 3.3.13. 4β-{4^''^-[1^'''^-(2^'''^,3^'''^,4^'''^,6^'''^-Tetra-*O*-butyryl-α-d-mannopyranosyloxy)-3,6,9-trioxadec-10-yl]-1,2,3-triazol-1-yl}-4-deoxypodophyllotoxin (**26a**)

White amorphous powder, yield 84% (after chromatography with petroleum ether/acetone, 1:1); mp 90 °C; ${\left[\text{α}\right]}_{D}^{26.9}$: −9.5 (c 0.26, CH_3_OH); ^1^H-NMR (CD_3_OD, 400 MHz) δ 7.79 (s, 1H, C^5^^''^-H), 6.69 (s, 1H, C^5^-H), 6.62 (s, 1H, C^8^-H), 6.40 (s, 2H, C^2^^'^, C^6^^'^-CH), 6.26 (d, 1H, *J* = 4.8 Hz, C^4^-H), 5.97 (d, 2H, *J* = 4.4 Hz, OCH_2_O), 5.34 (d, 1H, *J* = 8.0 Hz, C^4^^'''^-H), 5.29–5.26 (m, 3H, C^1^^'''^-H, C^3^^'''^-H, C^2^^'''^-H), 4.88–4.87 (m, 2H), 4.79 (d, 1H, *J* = 4.8 Hz, C^1^-H), 4.40–4.36 (m, 1H), 4.23 (dd, 1H, *J* = 4.8 Hz, 10.8 Hz), 4.13–4.10 (m, 2H), 3.73 (s, 6H, C^3^^'^, C^5^^'^-OCH_3_), 3.72 (s, 3H, C^4^^'^-OCH_3_), 3.65–3.60 (m, 12H, 3 × OCH_2_CH_2_O), 3.42 (dd, 1H, *J* = 4.8 Hz, 10.0 Hz, C^2^-H), 3.18–3.13 (m, 1H, C^3^-H), 2.39 (t, 2H, *J* = 7.2 Hz, COCH_2_), 2.32 (t, 2H, *J* = 7.2 Hz, COCH_2_), 2.26 (t, 2H, *J* = 7.2 Hz, COCH_2_), 2.18 (t, 2H, *J* = 7.2 Hz, COCH_2_), 1.71–1.62 (m, 4H, 2 × C*H*_2_CH_3_), 1.61–1.51 (m, 4H, 2 × C*H*_2_CH_3_), 0.99–0.88 (m, 12H, 4 × CH_2_C*H*_3_); ^13^C-NMR (CD_3_OD, 100 MHz) δ 175.7 (C-12), 174.7 (C=O), 174.0 (C=O), 173.9 (C=O), 173.8 (C=O), 154.0 (C-3^'^, C-5^'^), 150.6 (C-7), 149.3 (C-6), 146.1 (C-4^''^), 140.6 (C-1^'^), 138.3 (C-9), 134.8 (C-10), 127.0 (C-4^'^), 125.8 (C-5^''^), 111.2 (C-5), 109.9 (C-8), 109.4 (C-2^'^, C-6^'^), 103.3 (OCH_2_O), 99.0 (C-1^'''^), 71.6, 71.5, 71.4, 71.2, 70.9, 70.8, 70.5, 69.8, 68.9 (C-11), 68.3, 66.8, 65.1 (C-6^''^), 63.1 (C-6^'''^), 61.1 (4^'^-OCH_3_), 59.8 (C-2), 56.6 (3^'^, 5^'^-OCH_3_), 44.9 (C-4), 42.5 (C-1), 38.6 (C-3), 36.9 (CO*C*H_2_), 36.8 (CO*C*H_2_), 36.8 (CO*C*H_2_), 36.8 (CO*C*H_2_), 19.6 (*C*H_2_CH_3_), 19.4 (*C*H_2_CH_3_), 19.4 (*C*H_2_CH_3_), 19.2 (*C*H_2_CH_3_), 14.1 (CH_2_*C*H_3_), 14.0 (CH_2_*C*H_3_), 14.0 (CH_2_*C*H_3_), 14.0 (CH_2_*C*H_3_); ESIMS: *m/z* 1092 [M+Na]^+^, HRESIMS: calcd for C_53_H_71_N_3_O_20_H [M+H]^+^ 1070.4704, found 1070.4677.

#### 3.3.14. 4β-{4^''^-[1^'''^-(2^'''^,3^'''^,4^'''^,6^'''^-Tetra-*O*-butyryl-β-d-mannopyranosyloxy)-3,6,9-trioxadec-10-yl]-1,2,3-triazol-1-yl}-4-deoxypodophyllotoxin (**26b**)

White amorphous powder, yield 85% (after chromatography with petroleum ether/acetone, 1:1); mp 74–75 °C; ${\left[\text{α}\right]}_{D}^{25.7}$: −16.1 (c 0.21, CH_3_OH); ^1^H-NMR (CD_3_OD, 400 MHz) δ 7.80 (s, 1H, C^5^^''^-H), 6.69 (s, 1H, C^5^-H), 6.63 (s, 1H, C^8^-H), 6.41 (s, 2H, C^2^^'^, C^6^^'^-H), 6.26 (d, 1H, *J* = 4.8 Hz, C^4^-H), 5.97 (d, 2H, *J* = 4.8 Hz, OCH_2_O), 5.22 (d, 1H, *J* = 1.6 Hz, C^2^^'''^-H), 5.12 (dd, 1H, *J* = 4.0 Hz, 10.0 Hz, C^3^^'''^-H), 4.80–4.79 (m, 2H), 4.66 (s, 1H), 4.43–4.36 (m, 3H), 4.26 (dd, 1H, *J* = 4.0 Hz, 10.0 Hz), 3.94–3.90 (m, 1H), 3.73 (s, 6H, C^3^^'^, C^5^^'^-OCH_3_), 3.72 (s, 3H, C^4^^'^-OCH_3_), 3.65–3.60 (m, 12H, 3 × OCH_2_CH_2_O), 3.43 (dd, 1H, *J* = 4.0 Hz, 10.0 Hz, C^2^-H), 3.18–3.13 (m, 1H, C^3^-H), 2.35–2.25 (m, 8H, 4 × COCH_2_), 1.68–1.58 (m, 8H, 4 × C*H*_2_CH_3_), 0.97–0.92 (m, 12H, 4 × CH_2_C*H*_3_); ^13^C-NMR (CD_3_OD, 100 MHz) δ 179.0 (C-12), 175.8 (C=O), 175.0 (C=O), 174.4 (C=O), 174.0 (C=O), 154.7 (C-4^''^), 154.0 (C-3^'^, C-5^'^), 150.6 (C-7), 149.3 (C-6), 138.3 (C-1^'^), 136.8 (C-9), 134.8 (C-10), 127.0 (C-4^'^), 125.9 (C-5^''^), 110.2 (C-5), 109.9 (C-8), 109.4 (C-2^'^, C-6^'^), 103.3 (OCH_2_O), 99.0 (C-1^'''^), 73.1, 72.1, 71.6, 71.5, 71.4, 71.2, 70.9, 70.7, 68.2 (C-11), 66.0, 65.0 (C-6^''^), 64.2 (C-6^'''^), 61.1 (4^'^-OCH_3_), 59.8 (C-2), 56.6 (3^'^, 5^'^-OCH_3_), 44.9 (C-4), 42.5 (C-1), 38.6 (C-3), 37.0 (CO*C*H_2_), 36.9 (CO*C*H_2_), 36.9 (CO*C*H_2_), 36.8 (CO*C*H_2_), 19.6 (*C*H_2_CH_3_), 19.5 (*C*H_2_CH_3_), 19.4 (*C*H_2_CH_3_), 19.2 (*C*H_2_CH_3_), 14.1 (CH_2_*C*H_3_), 14.0 (CH_2_*C*H_3_), 14.0 (CH_2_*C*H_3_), 14.0 (CH_2_*C*H_3_); ESIMS: *m/z* 1092 [M+Na]^+^, HRESIMS: calcd for C_53_H_71_N_3_O_20_H [M+H]^+^ 1070.4704, found 1070.4703.

#### 3.3.15. 4β-{4^''^-[1^'''^-(2^'''^,3^'''^,4^'''^,6^'''^-Tetra-*O*-butyryl-α-d-mannopyranosyloxy)-3,6,9-trioxadec-10-yl]-1,2,3-triazol-1-yl}-4-deoxy-4^'^-demethylpodophyllotoxin (**27a**)

White amorphous powder, yield 89% (after chromatography with petroleum ether/acetone, 1:1); mp 95–97 °C; ${\left[\text{α}\right]}_{D}^{26.7}$: −12.6 (c 0.29, CH_3_OH); ^1^H-NMR (CD_3_OD, 400 MHz) δ 7.79 (s, 1H, C^5^^''^-H), 6.69 (s, 1H, C^5^-H), 6.65 (s, 1H, C^8^-H), 6.38 (s, 2H, C^2^^'^, C^6^^'^-H), 6.26 (d, 1H, *J* = 4.8 Hz, C^4^-H), 5.98 (d, 2H, *J* = 5.2 Hz, OCH_2_O), 5.34 (d, 1H, *J* = 10.0 Hz, C^4^^'''^-H), 5.28–5.26 (m, 2H, C^3^^'''^-H, C^2^^'''^-H), 4.88–4.87 (m, 2H), 4.76 (d, 1H, *J* = 4.4 Hz, C^1^-H), 4.41–4.37 (m, H), 4.23 (dd, 1H, *J* = 4.8 Hz, 10.8 Hz), 4.23–4.10 (m, 2H), 3.83–3.78 (m, 1H), 3.74 (s, 6H, C^3^^'^-OCH_3_, C^5^^'^-OCH_3_), 3.66–3.61 (m, 12H, 3 × OCH_2_CH_2_O), 3.39 (dd, 1H, *J* = 4.8 Hz, 10.0 Hz, C^2^-H), 3.18–3.13 (m, 1H, C^3^-H), 2.39 (t, 2H, *J* = 7.6 Hz, COCH_2_), 2.32 (t, 2H, *J* = 7.6 Hz, COCH_2_), 2.26 (t, 2H, *J* = 7.6 Hz, COCH_2_), 2.18 (t, 2H, *J* = 7.6 Hz, COCH_2_), 1.71–1.62 (m, 4H, 2 × C*H*_2_CH_3_), 1.61–1.51 (m, 4H, 2 × C*H*_2_CH_3_), 0.99–0.88 (t, 12H, 4 × CH_2_C*H*_3_); ^13^C-NMR (CD_3_OD, 100 MHz) δ 176.0 (C-12), 174.8 (C=O), 174.0 (C=O), 173.9 (C=O), 173.8 (C=O), 150.5 (C-7), 149.2 (C-6), 148.7 (C-3^'^, C-5^'^), 146.1 (C-4^''^), 136.0 (C-1^'^), 135.2 (C-9), 131.3 (C-10), 127.0 (C-4^'^), 125.8 (C-5^''^), 111.3 (C-5), 109.8 (C-8), 109.3 (C-2^'^, C-6^'^), 103.3 (OCH_2_O), 99.0 (C-1^'''^), 71.6, 71.5, 71.4, 71.2, 70.9, 70.8, 70.5, 69.8, 68.9 (C-11), 68.3, 66.8, 65.1 (C-6^''^), 63.1 (C-6^'''^), 59.9 (C-2), 56.8 (3^'^, 5^'^-OCH_3_), 44.8 (C-4), 42.8 (C-1), 38.5 (C-3), 36.9 (CO*C*H_2_), 36.9 (CO*C*H_2_), 36.8 (CO*C*H_2_), 36.8 (CO*C*H_2_), 19.6 (*C*H_2_CH_3_), 19.3 (*C*H_2_CH_3_), 19.3 (*C*H_2_CH_3_), 19.2 (*C*H_2_CH_3_), 14.1 (CH_2_*C*H_3_), 14.0 (CH_2_*C*H_3_), 14.0 (CH_2_*C*H_3_), 13.9 (CH_2_*C*H_3_); ESIMS: *m/z* 1078 [M+Na]^+^, HRESIMS: calcd for C_52_H_69_N_3_O_20_H [M+H]^+^ 1056.4547, found 1056.4533.

#### 3.3.16. 4β-{4^''^-[1^'''^-(2^'''^,3^'''^,4^'''^,6^'''^-Tetra-*O*-butyryl-β-d-mannopyranosyloxy)-3,6,9-trioxadec-10-yl]-1,2,3-triazol-1-yl}-4-deoxy-4^'^-demethylpodophyllotoxin (**27b**)

White amorphous powder, yield 88% (after chromatography with petroleum ether/acetone, 1:1); mp 80–82 °C; ${\left[\text{α}\right]}_{D}^{25.8}$: −26.6 (c 0.25, CH_3_OH); ^1^H-NMR (CD_3_OD, 400 MHz) δ 7.80 (s, 1H, C^5^^''^-H), 6.67 (s, 1H, C^5^-H), 6.62 (s, 1H, C^8^-H), 6.41 (s, 2H, C^2^^'^, C^6^^'^-H), 6.24 (d, 1H, *J* = 4.8 Hz, C^4^-H), 5.97 (d, 2H, *J* = 5.6 Hz, OCH_2_O), 5.23 (d, 1H, *J* = 1.6 Hz), 5.12 (dd, 1H, *J* = 4.0 Hz, 10.0 Hz, C^3^^'''^-H), 4.80 (d, 1H, *J* = 1.6 Hz), 4.76 (d, 1H, *J* = 4.0 Hz), 4.43–4.35 (m, 3H), 4.26 (dd, 1H, *J* = 1.2 Hz, 5.2 Hz), 3.94–3.90 (m, 1H), 3.82–3.80 (m, 1H), 3.74 (s, 6H, C^3^^'^, C^5^^'^-OCH_3_), 3.65–3.60 (m, 12H, 3 × OCH_2_CH_2_O), 3.39 (dd, 1H, *J* = 4.0 Hz, 10.0 Hz, C^2^-H), 3.16–3.12 (m, 1H, C^3^-H), 2.35–2.24 (m, 8H, 4 × COCH_2_), 1.68–1.58 (m, 8H, 4 × C*H*_2_CH_3_), 0.97–0.91 (m, 12H, 4 × CH_2_C*H*_3_); ^13^C-NMR (CD_3_OD, 100 MHz) δ 179.0 (C-12), 175.9 (C=O), 175.1 (C=O), 174.5 (C=O), 174.0 (C=O), 150.5 (C-7), 149.2 (C-6), 148.7 (C-3^'^, C-5^'^), 146.1 (C-4^''^), 136.0 (C-1^'^), 135.1 (C-9), 131.4 (C-10), 126.9 (C-4^'^), 125.9 (C-5^''^), 111.3 (C-5), 109.8 (C-8), 109.4 (C-2^'^, C-6^'^), 103.3 (OCH_2_O), 99.0 (C-1^'''^), 73.1, 72.1, 71.6, 71.5, 71.5, 71.2, 70.9, 70.7, 68.9 (C-11), 68.2, 66.0, 65.0, (C-6^''^), 64.2 (C-6^'''^), 59.9 (C-2), 56.8 (3^'^, 5^'^-OCH_3_), 44.8 (C-4), 42.7 (C-1), 38.5 (C-3), 37.0 (CO*C*H_2_), 36.9 (CO*C*H_2_), 36.9 (CO*C*H_2_), 36.9 (CO*C*H_2_), 19.6 (*C*H_2_CH_3_), 19.5 (*C*H_2_CH_3_), 19.4 (*C*H_2_CH_3_), 19.2 (*C*H_2_CH_3_), 14.0 (CH_2_*C*H_3_), 14.0 (CH_2_*C*H_3_), 14.0 (CH_2_*C*H_3_), 14.0 (CH_2_*C*H_3_); ESIMS: *m/z* 1078 [M+Na]^+^, HRESIMS: calcd for C_52_H_69_N_3_O_20_H [M+H]^+^ 1056.4547, found 1056.4509.

#### 3.3.17. 4β-{4^''^-[1^"'^-(2^"'^,3^"'^,4^"'^-Tri-*O*-butyryl-α-d-xylopyranosyloxy)-1,2,3-triazol-1-yl]}-4-deoxy-podophyllotoxin (**28a**)

White amorphous powder, yield 83% (after chromatography with petroleum ether/acetone, 1:1); mp 98–99 °C; ${\left[\text{α}\right]}_{D}^{26.3}$: +20.5 (c 0.26, CH_3_OH); ^1^H-NMR (CD_3_OD, 400 MHz) δ 7.86 (s, 1H, C^5^^''^-H), 6.68 (s, 1H, C^5^-H), 6.59 (s, 1H, C^8^-H), 6.41 (s, 2H, C^2^^'^, C^6^^'^-H), 6.24 (d, 1H, *J* = 4.4 Hz, C^4^-H), 5.96 (d, 2H, *J* = 8.0 Hz, OCH_2_O), 5.44 (t, 1H, *J* = 10.0 Hz, C^3^^"'^-H), 5.13 (d, 1H, *J* = 3.2 Hz, C^1^^"'^-H), 5.00–5.96 (m, 1H, C^2^^"'^-H), 4.77–4.73 (m, 3H), 4.65 (d, 1H, *J* = 4.8 Hz, C^1^-H), 4.38–4.35 (m, 1H), 3.80–3.78 (m, 3H), 3.73 (s, 6H, C^3^^'^, C^5^^'^-OCH_3_), 3.71 (s, 3H, C^4^^'^-OCH_3_), 3.43 (dd, 1H, *J* = 4.8 Hz, 10.4 Hz, C^2^-H), 3.19–3.14 (m, 1H, C^3^-H), 2.24–2.17 (m, 6H, 3 × COCH_2_), 1.60–1.49 (m, 6H, 3 × C*H*_2_CH_3_), 0.90–0.88 (m, 9H, 3 × CH_2_C*H*_3_); ^13^C-NMR (CD_3_OD, 100 MHz) δ 175.7 (C-12), 174.1 (C=O), 174.0 (C=O), 173.9 (C=O), 154.0 (C-3^'^, C-5^'^), 150.6 (C-7), 149.3 (C-6), 144.6 (C-4^''^), 138.3 (C-1^'^), 136.7 (C-9), 134.7 (C-10), 127.0 (C-4^'^), 126.4 (C-5^''^), 111.2 (C-5), 109.9 (C-8), 109.4 (C-2^'^, C-6^'^), 103.3 (OCH_2_O), 96.1 (C-1^'''^), 72.2, 70.5, 70.3, 68.9 (C-11), 61.2 (C-5^'''^), 61.1 (4^'^-OCH_3_), 59.8 (C-2), 59.6 (C-6^''^), 56.6 (3^'^, 5^'^-OCH_3_), 44.9 (C-4), 42.5 (C-1), 38.6 (C-3), 36.8 (CO*C*H_2_), 36.7 (CO*C*H_2_), 36.7 (CO*C*H_2_), 19.4 (*C*H_2_CH_3_), 19.4 (*C*H_2_CH_3_), 19.3 (*C*H_2_CH_3_), 14.0 (CH_2_*C*H_3_), 13.9 (CH_2_*C*H_3_), 13.9 (CH_2_*C*H_3_); ESIMS: *m/z* 861 [M+Na]^+^, HRESIMS: calcd for C_42_H_51_N_3_O_25_H [M+H]^+^ 838.3393, found 838.3367.

#### 3.3.18. 4β-{4^''^-[1^'''^-(2^'''^,3^'''^,4^'''^-Tri-*O*-butyryl-β-d-xylopyranosyloxy)-1,2,3-triazol-1-yl]}-4-deoxy-podophyllotoxin (**28b**)

White amorphous powder, yield 83% (after chromatography with petroleum ether/acetone, 1:1); mp 97–99 °C; ${\left[\text{α}\right]}_{D}^{26.7}$: −99.9 (c 0.25, Pyridine); ^1^H-NMR (C_5_D_5_N, 500 MHz) δ 8.14 (s, 1H, C^5^^''^-H), 6.86 (s, 1H, C^5^-H), 6.86 (s, 1H, C^8^-H), 6.76 (s, 2H, C^2^^'^, C^6^^'^-H), 6.57 (d, 1H, *J* = 5.0 Hz, C^4^-H), 6.00 (d, 2H, *J* = 10.0 Hz, OCH_2_O), 5.67 (d, 1H, *J* = 9.0 Hz), 5.42 (t, 1H, *J* = 9.0 Hz, C^3^^'''^-H), 5.33–5.29 (m, 1H), 5.14–5.12 (m, 2H), 5.10 (d, 1H, *J* = 8.0 Hz, C^1^^'''^-H), 5.01 (d, 1H, *J* = 5.0 Hz, C^1^-H), 5.06–5.04 (m, 2H), 4.42 (t, 1H, *J* = 8.0 Hz), 4.28 (dd, 1H, *J* = 5.0 Hz, 10.0 Hz), 3.82 (s, 6H, C^3^^'^, C^5^^'^-OCH_3_), 3.78 (s, 3H, C^4^^'^-OCH_3_), 3.60–3.58 (m, 1H, C^2^-H), 3.45–3.42 (m, 1H, C^3^-H), 2.30–2.24 (m, 6H, 3 × COCH_2_) 1.60–1.54 (m, 6H, 3 × C*H*_2_CH_3_), 0.83–0.78 (m, 9H, 3 × C*H*_2_CH_3_); ^13^C-NMR (C_5_D_5_N, 100 MHz) δ 174.0 (C-12), 172.6 (C=O), 172.6 (C=O), 172.3 (C=O), 153.5 (C-3^'^, C-5^'^), 149.5 (C-7), 148.3 (C-6), 144.6 (C-4^''^), 138.3 (C-1^'^), 136.7 (C-9), 134.0 (C-10), 126.4 (C-4^'^), 124.9 (C-5^''^), 110.7 (C-5), 109.4 (C-8), 109.2 (C-2^'^, C-6^'^), 102.5 (OCH_2_O), 100.5 (C-1^'''^), 72.1, 71.4, 69.4, 67.9 (C-11), 62.7 (C-5^'''^), 62.6 (C-6^''^), 60.6 (4^'^-OCH_3_), 58.8 (C-2), 56.2 (3^'^, 5^'^-OCH_3_), 44.3 (C-4), 41.9 (C-1), 38.0 (C-3), 36.0 (CO*C*H_2_), 36.0 (CO*C*H_2_), 35.9 (CO*C*H_2_), 18.7 (*C*H_2_CH_3_), 18.6 (*C*H_2_CH_3_), 18.6 (*C*H_2_CH_3_), 13.6 (CH_2_*C*H_3_), 13.6 (CH_2_*C*H_3_), 13.6 (CH_2_*C*H_3_); ESIMS: *m/z* 860 [M+Na]^+^, HRESIMS: calcd for C_42_H_51_N_3_O_15_H [M+H]^+^ 838.3393, found 838.3369.

#### 3.3.19. 4β-{4^''^-[1^'''^-(2^'''^,3^'''^,4^'''^-Tri-*O*-butyryl-α-d-xylopyranosyloxy)-1,2,3-triazol-1-yl]}-4-deoxy-4^'^-demethylpodophyllotoxin (**29a**)

White amorphous powder, yield 84% (after chromatography with petroleum ether/acetone, 1:1); mp 200–203 °C; ${\left[\text{α}\right]}_{D}^{26.6}$: −27.3 (c 0.25, Pyridine); ^1^H-NMR (C_5_D_5_N, 400 MHz) δ 8.30 (s, 1H, C^5^^''^-H), 6.87 (s, 1H, C^5^-H), 6.85 (s, 1H, C^8^-H), 6.81 (s, 2H, C^2^^'^, C^6^^'^-H), 6.56 (d, 1H, *J* = 4.8 Hz, C^4^-H), 6.03–5.96 (m, 3H, OCH_2_O, C^4^^'''^-H), 5.64 (d, 1H, *J* = 4.0 Hz, C^1^^'''^-H), 5.41–5.35 (m, 1H, C^3^^'''^-H), 5.23 (dd, 1H, *J* = 4.0 Hz, 10.0 Hz, C^2^^'''^-H), 5.08–5.05 (m, 2H), 5.02 (d, 1H, *J* = 5.0 Hz, C^1^-H), 4.45 (t, 1H, *J* = 8.0 Hz), 4.01–3.94 (m, 3H), 3.80 (dd, 1H, *J* = 5.0 Hz, 10.0 Hz, C^2^-H), 3.72 (s, 6H, C^3^^'^, C^5^^'^-OCH_3_), 3.46 (t, 1H, *J* = 10.0 Hz, C^3^-H), 2.32–2.20 (m, 6H, 3 × COCH_2_), 1.62–1.50 (m, 6H, 3 × C*H*_2_CH_3_), 0.83–0.77 (m, 9H, 3 × CH_2_C*H*_3_); ^13^C-NMR (C_5_D_5_N, 100 MHz) δ 174.1 (C-12), 172.8 (C=O), 172.7 (C=O), 172.6 (C=O), 149.4 (C-7), 148.8 (C-3^'^, C-5^'^), 148.2 (C-6), 144.0 (C-4^''^), 137.4 (C-1^'^), 134.5 (C-9), 130.0 (C-10), 126.4 (C-5^''^), 125.4 (C-4^'^), 110.7 (C-5), 109.7 (C-2^'^, C-6^'^), 109.3 (C-8), 102.4 (OCH_2_O), 95.4 (C-1^'''^), 71.5, 69.7, 69.6, 67.9 (C-11), 60.9 (C-5^'''^), 59.1 (C-2), 58.9 (C-6^''^), 56.5 (3^'^, 5^'^-OCH_3_), 44.2 (C-4), 42.1 (C-1), 37.9 (C-3), 36.1 (CO*C*H_2_), 36.0 (CO*C*H_2_), 35.9 (CO*C*H_2_), 18.7 (*C*H_2_CH_3_), 18.6 (*C*H_2_CH_3_), 18.6 (*C*H_2_CH_3_), 13.6 (CH_2_*C*H_3_), 13.6 (CH_2_*C*H_3_), 13.6 (CH_2_*C*H_3_); ESIMS: *m/z* 846 [M+Na]^+^, HRESIMS: calcd for C_41_H_49_N_3_O_15_H [M+H]^+^ 824.3236, found 824.3226.

#### 3.3.20. 4β-{4^''^-[1^'''^-(2^'''^,3^'''^,4^'''^-Tri-*O*-butyryl-β-d-xylopyranosyloxy)-1,2,3-triazol-1-yl]}-4-deoxy-4^'^-demethylpodophyllotoxin (**29b**)

White amorphous powder, yield 82% (after chromatography with petroleum ether/acetone, 1:1); mp 100–101 °C; ${\left[\text{α}\right]}_{D}^{26.7}$: −121.4 (c 0.19, Pyridine); ^1^H-NMR (C_5_D_5_N, 400 MHz) δ 8.14 (s, 1H, C^5^^''^-H), 6.87 (s, 1H, C^5^-H), 6.83 (s, 1H, C^8^-H), 6.80 (s, 2H, C^2^^'^, C^6^^'^-H), 6.56 (d, 1H, *J* = 4.8 Hz, C^4^-H), 6.00–5.97 (m, 2H, OCH_2_O), 5.68 (t, 1H, *J* = 8.8 Hz, C^3^^'''^-H), 5.46–5.42 (m, 1H, C^4^^'''^-H), 5.35–5.30 (m, 1H, C^2^^'''^-H), 5.15–5.14 (m, 2H), 5.11 (d, 1H, *J* = 7.2 Hz, C^1^^'''^-H), 5.00–4.99 (m, 3H), 4.46 (t, 1H, *J* = 8.0 Hz), 4.29 (dd, 1H, *J* = 5.0 Hz, 10.0 Hz), 3.72 (s, 6H, C^3^^'^, C^5^^'^-OCH_3_), 3.67–3.58 (m, 1H, C^2^-H), 3.44–3.42 (m, 1H, C^3^-H), 2.31–2.21 (m, 6H, 3 × COCH_2_), 1.61–1.52 (m, 6H, 3 × C*H*_2_CH_3_), 0.85–0.78 (m, 9H, 3 × CH_2_C*H*_3_); ^13^C-NMR (C_5_D_5_N, 100 MHz) δ 174.1 (C-12), 172.7 (C=O), 172.6 (C=O), 172.3 (C=O), 149.5 (C-7), 148.8 (C-3^'^, C-5^'^), 148.2 (C-6), 144.6 (C-4^''^), 137.4 (C-1^'^), 134.5 (C-9), 130.0 (C-10), 126.3 (C-5^''^), 124.9 (C-4^'^), 110.8 (C-5), 109.7 (C-2^'^, C-6^'^), 109.3 (C-8), 102.4 (OCH_2_O), 100.5 (C-1^'''^), 72.1, 71.4, 69.4, 67.9 (C-11), 62.7 (C-5^'''^), 62.6 (C-6^''^), 58.9 (C-2), 56.5 (3^'^, 5^'^-OCH_3_), 44.1 (C-4), 42.1 (C-1), 38.0 (C-3), 36.1 (CO*C*H_2_), 36.0 (CO*C*H_2_), 35.9 (CO*C*H_2_), 18.7 (*C*H_2_CH_3_), 18.6 (*C*H_2_CH_3_), 18.6 (*C*H_2_CH_3_), 13.6 (CH_2_*C*H_3_), 13.6 (CH_2_*C*H_3_), 13.6 (CH_2_*C*H_3_); ESIMS: *m/z* 860 [M+Na]^+^, HRESIMS: calcd for C_42_H_51_N_3_O_15_H [M+H]^+^ 838.3393, found 838.3369.

#### 3.3.21. 4β-{4^''^-[1^'''^-(2^'''^,3^'''^,4^'''^-Tri-*O*-butyryl-α-d-xylopyranosyloxy)-3,6,9-trioxadec-10-yl]-1,2,3-triazol-1-yl}-4-deoxypodophyllotoxin (**30a**)

White amorphous power, yield 83% (after chromatography with petroleum ether/acetone, 1:1); mp 84 °C; ${\left[\text{α}\right]}_{D}^{26.5}$: +12.2 (c 0.28, CH_3_OH); ^1^H-NMR (CD_3_OD, 400 MHz) δ 7.78 (s, 1H, C^5^^''^-H), 6.68 (s, 1H, C^5^-H), 6.60 (s, 1H, C^8^-H), 6.41 (s, 2H, C^2^^'^, C^6^^'^-H), 6.25 (d, 1H, *J* = 4.8 Hz, C^4^-H), 5.97 (d, 2H, *J* = 5.2 Hz, OCH_2_O), 5.47 (t, 1H, *J* = 10.0 Hz, C^3^^'''^-H), 5.06–5.04 (m, 1H, C^4^^'''^-H), 4.96–4.94 (m, 1H, C^2^^'''^-H), 4.84 (d, 1H, *J* = 4.0 Hz, C^1^^'''^-H), 4.81 (d, 1H, *J* = 4.0 Hz, C^1^-H), 4.79–4.78 (m, 2H), 4.39–4.34 (m, 1H), 3.80–3.78 (m, 3H), 3.73 (s, 6H, C^3^^'^, C^5^^'^-OCH_3_), 3.71 (s, 3H, C^4^^'^-OCH_3_), 3.65–3.59 (m, 12H, 3 × OCH_2_CH_2_O), 3.41 (dd, 1H, *J* = 4.0 Hz, 10.8 Hz, C^2^-H), 3.17–3.12 (m, 1H, C^3^-H), 2.27–2.23 (m, 6H, 3 × COCH_2_), 1.60–1.53 (m, 6H, 3 × C*H*_2_CH_3_), 0.92–0.87 (m, 9H, 3 × CH_2_C*H*_3_); ^13^C-NMR (CD_3_OD, 100 MHz) δ 175.7 (C-12), 174.1 (C=O), 174.1 (C=O), 173.9 (C=O), 154.0 (C-3^'^, C-5^'^), 150.5 (C-7), 149.3 (C-6), 146.1 (C-4^''^), 138.3 (C-1^'^), 136.7 (C-9), 134.8 (C-10), 127.0 (C-4^'^), 125.8 (C-5^''^), 111.2 (C-5), 109.9 (C-8), 109.4 (C-2^'^, C-6^'^), 103.3 (OCH_2_O), 97.3 (C-1^'''^), 72.2, 71.7, 71.6, 71.5, 71.3, 71.0, 70.7, 70.5, 68.9 (C-11), 68.5, 65.1 (C-5^'''^), 61.2 (4^'^-OCH_3_), 59.8 (C-2), 59.4 (C-6^''^), 56.7 (3^'^, 5^'^-OCH_3_), 44.9 (C-4), 42.5 (C-1), 38.6 (C-3), 36.9 (CO*C*H_2_), 36.8 (CO*C*H_2_), 36.6 (CO*C*H_2_), 19.4 (*C*H_2_CH_3_), 19.4 (*C*H_2_CH_3_), 19.3 (*C*H_2_CH_3_), 14.0 (CH_2_*C*H_3_), 14.0 (CH_2_*C*H_3_), 14.0 (CH_2_*C*H_3_); ESIMS: *m/z* 992 [M+Na]^+^, HRESIMS: calcd for C_48_H_63_N_3_O_18_H [M+H]^+^ 970.4179, found 970.4167.

#### 3.3.22. 4β-{4^''^-[1^"'^-(2^"'^,3^"'^,4^"'^-Tri-*O*-butyryl-β-d-xylopyranosyloxy)-3,6,9-trioxadec-10-yl]-1,2,3-triazol-1-yl}-4-deoxypodophyllotoxin (**30b**)

White amorphous powder, yield 85% (after chromatography with petroleum ether/acetone, 1:1); mp 88–90 °C; ${\left[\text{α}\right]}_{D}^{26.5}$: −45.9 (c 0.22, CH_3_OH); ^1^H-NMR (CD_3_OD, 400 MHz) δ 7.80 (s, 1H, C^5^^''^-H), 6.69 (s, 1H, C^5^-H), 6.62 (s, 1H, C^8^-H), 6.42 (s, 2H, C^2^^'^, C^6^^'^-H), 6.26 (d, 1H, *J* = 4.8 Hz, C^4^-H), 5.97 (d, 2H, *J* = 5.6 Hz, OCH_2_O), 5.24 (t, 1H, *J* = 9.2 Hz, C^3^^"'^-H), 4.89–4.86 (m, 2H, C^2^^"'^-H, C^4^^"'^-H), 4.80 (d, 1H, *J* = 5.2 Hz, C^1^-H), 4.64 (d, 1H, *J* = 8.0 Hz, C^1^^"'^-H), 4.63–4.62 (m, 2H), 4.41–4.36 (m, 1H), 4.06–4.02 (m, 1H), 3.88–3.83 (m, 1H), 3.81–3.79 (s, 2H, C^5^^'''^-CH_2_), 3.74 (s, 6H, C^3^^'^, C^5^^'^-OCH_3_), 3.72 (s, 3H, C^4^^'^-OCH_3_), 3.66–3.3.60 (m, 12H, 3 × OCH_2_CH_2_O), 3.48–3.43 (m, 1H, C^2^-H), 3.18–3.14 (m, 1H, C^3^-H), 2.30–2.20 (m, 6H, 3 × COCH_2_), 1.61–1.53 (m, 6H, 3 × C*H*_2_CH_3_), 0.91–0.89 (m, 9H, 3 × CH_2_C*H*_3_); ^13^C-NMR (CD_3_OD, 100 MHz) δ 175.8 (C-12), 174.0 (C=O), 174.0 (C=O), 173.7 (C=O), 154.0 (C-3^'^, C-5^'^), 150.6 (C-7), 149.3 (C-6),146.1 (C-4^''^), 138.3 (C-1^'^), 136.7 (C-9), 134.8 (C-10), 127.0 (C-4^'^), 125.8 (C-5^''^), 111.2 (C-5), 109.9 (C-8), 109.4 (C-2^'^, C-6^'^), 103.3 (OCH_2_O), 102.3 (C-1^'''^), 73.1, 72.3, 71.6, 71.5, 71.4, 70.9, 70.3, 69.9 (C-11), 65.1 (C-5^'''^), 63.3 (C-6^''^), 61.6 (4^'^-OCH_3_), 59.8 (C-2), 56.6 (3^'^, 5^'^-OCH_3_), 44.9 (C-4), 42.5 (C-1), 38.6 (C-3), 36.9 (CO*C*H_2_), 36.8 (CO*C*H_2_), 36.7 (CO*C*H_2_), 19.4 (*C*H_2_CH_3_), 19.4 (*C*H_2_CH_3_), 19.3 (*C*H_2_CH_3_), 14.0 (CH_2_*C*H_3_), 14.0 (CH_2_*C*H_3_), 13.9 (CH_2_*C*H_3_); ESIMS: *m/z* 992 [M+Na]^+^, HRESIMS: calcd for C_48_H_63_N_3_O_18_H [M+H]^+^ 970.4179, found 970.4162.

#### 3.3.23. 4β-{4^''^-[1^'''^-(2^'''^,3^'''^,4^'''^-Tri-*O*-butyryl-α-d-xylopyranosyloxy)-3,6,9-trioxadec-10-yl]-1,2,3-triazol-1-yl}-4-deoxy-4^'^-demethylpodophyllotoxin (**31a**)

White amorphous powder, yield 86% (after chromatography with petroleum ether/acetone, 1:1); mp 87–88 °C; ${\left[\text{α}\right]}_{D}^{26.2}$: +7.1 (c 0.22, CH_3_OH); ^1^H-NMR (CD_3_OD, 400 MHz) δ 7.77 (s, 1H, C^5^^''^-H), 6.67 (s, 1H, C^5^-H), 6.63 (s, 1H, C^8^-H), 6.38 (s, 2H, C^2^^'^, C^6^^'^-H), 6.24 (d, 1H, *J* = 4.4 Hz, C^4^-H), 5.97 (d, 2H, *J* = 5.6 Hz, OCH_2_O), 5.47 (t, 1H, *J* = 10.0 Hz, C^3^^'''^-H), 5.06 (d, 1H, *J* = 3.2 Hz, C^1^^'''^-H), 4.97–4.94 (m, 1H, C^2^^'''^-H), 4.85–4.84 (m, 1H, C^4^^'''^-H), 4.81 (d, 1H, *J* = 4.0 Hz, C^1^-H), 4.76–4.73 (m, 2H), 4.37 (t, 1H, *J* = 7.2 Hz), 3.74 (s, 6H, C^3^^'^, C^5^^'^-OCH_3_), 3.66–3.60 (m, 12H, 3 × OCH_2_CH_2_O), 3.39 (dd, 1H, *J* = 4.0 Hz, 10.0 Hz, C^2^-H), 3.15 (t, 1H, *J* = 10.0 Hz, C^3^-H), 2.29–2.22 (m, 6H, 3 × COCH_2_), 1.61–1.54 (m, 6H, 3 × C*H*_2_CH_3_), 0.91–0.87 (m, 9H, 3 × CH_2_C*H*_3_); ^13^C-NMR (CD_3_OD, 100 MHz) δ 174.4 (C-12), 172.6 (C=O), 172.5 (C=O), 172.4 (C=O), 149.0 (C-7), 147.7 (C-6), 147.2 (C-3^'^, C-5^'^), 144.6 (C-4^''^), 134.5 (C-1^'^), 133.6 (C-9), 129.8 (C-10), 125.3 (C-4^'^), 124.2 (C-5^''^), 109.7 (C-5), 108.3 (C-8), 107.8 (C-2^'^, C-6^'^), 101.7 (OCH_2_O), 95.7 (C-1^'''^), 70.6, 70.1, 70.0, 69.7, 69.4, 69.1, 69.0, 67.4 (C-11), 67.0, 63.5 (C-5^'''^), 58.3 (C-2), 57.8 (C-6^''^), 55.2 (3^'^, 5^'^-OCH_3_), 43.2 (C-4), 41.2 (C-1), 37.0 (C-3), 35.3 (CO*C*H_2_), 35.2 (CO*C*H_2_), 35.1 (CO*C*H_2_), 17.9 (*C*H_2_CH_3_), 17.8 (*C*H_2_CH_3_), 17.8 (*C*H_2_CH_3_), 12.4 (CH_2_*C*H_3_), 12.4 (CH_2_*C*H_3_), 12.4 (CH_2_*C*H_3_); ESIMS: *m/z* 978 [M+Na]^+^, HRESIMS: calcd for C_47_H_61_N_3_O_18_H [M+H]^+^ 956.4023, found 956.4015.

#### 3.3.24. 4β-{4^''^-[1^'''^-(2^'''^,3^'''^,4^'''^-Tri-*O*-butyryl-β-d-xylopyranosyloxy)-3,6,9-trioxadec-10-yl]-1,2,3-triazol-1-yl}-4-deoxy-4^'^-demethylpodophyllotoxin (**31b**)

White amorphous powder, yield 87% (after chromatography with petroleum ether/acetone, 1:1); mp 79–80 °C; ${\left[\text{α}\right]}_{D}^{26.6}$: −93.4 (c 0.29, Pyridine); ^1^H-NMR (C_5_D_5_N, 400 MHz) δ 8.11 (s, 1H, C^5^^''^-H), 6.86 (s, 1H, C^5^-H), 6.85 (s, 1H, C^8^-H), 6.80 (s, 2H, C^2^^'^, C^6^^'^-H), 6.53 (d, 1H, *J* = 4.8 Hz, C^4^-H), 5.97 (d, 2H, *J* = 6.8 Hz, OCH_2_O), 5.69 (t, 1H, *J* = 9.2 Hz, C^3^^'''^-H), 5.44–5.40 (m, 1H, C^4^^'''^-H), 5.35–5.30 (m, 1H, C^2^^'''^-H), 4.98 (d, 1H, *J* = 4.8 Hz, C^1^-H), 4.87 (d, 1H, *J* = 7.2 Hz, C^1^^'''^-H), 4.84 (s, 2H), 4.44 (t, 1H, *J* = 8.0 Hz), 4.28 (dd, 1H, *J* = 6.0 Hz, 10.0 Hz), 4.01–3.96 (m, 1H), 3.78–3.75 (m, 1H), 3.72 (s, 6H, C^3^^'^, C^5^^'^-OCH_3_), 3.63–3.57 (m, 12H, 3 × OCH_2_CH_2_O), 3.45–3.41 (m, 1H, C^3^-H), 2.38–2.26 (m, 6H, 3 × COCH_2_), 1.66–1.52 (m, 6H, 3 × C*H*_2_CH_3_), 0.87–0.80 (m, 9H, 3 × CH_2_C*H*_3_); ^13^C-NMR (C_5_D_5_N, 100 MHz) δ 174.1 (C-12), 172.7 (C=O), 172.6 (C=O), 172.3 (C=O), 148.7 (C-7), 148.2 (C-3^'^, C-5^'^), 148.2 (C-6), 145.7 (C-4^''^), 137.4 (C-1^'^), 134.5 (C-9), 130.0 (C-10), 126.4 (C-4^'^), 124.5 (C-5^''^), 110.7 (C-5), 109.7 (C-2^'^, C-6^'^), 109.3 (C-8), 102.4 (OCH_2_O), 101.5 (C-1^'''^), 72.2, 71.5, 70.8, 70.7, 70.5, 70.4, 69.5, 69.0, 67.9 (C-11), 65.0 (C-5^'''^), 62.6 (C-2), 58.8 (C-6^''^), 56.5 (3^'^, 5^'^-OCH_3_), 44.1 (C-4), 42.1 (C-1), 37.9 (C-3), 36.1 (CO*C*H_2_), 36.1 (CO*C*H_2_), 36.0 (CO*C*H_2_), 18.7 (*C*H_2_CH_3_), 18.7 (*C*H_2_CH_3_), 18.6 (*C*H_2_CH_3_), 13.7 (CH_2_*C*H_3_), 13.7 (CH_2_*C*H_3_), 13.6 (CH_2_*C*H_3_); ESIMS: *m/z* 978 [M+Na]^+^, HRESIMS: calcd for C_47_H_61_N_3_O_18_H [M+H]^+^ 956.4023, found 956.4007.

### 3.4. Cell Culture and Cytotoxicity Assay

The following human tumor cell lines were used: HL-60, SMMC-7721, A-549, MCF-7, and SW480. All the cells were cultured in RMPI-1640 or DMEM medium (Hyclone, Logan, UT, USA), supplemented with 10% fetal bovine serum (Hyclone) at 37 °C in a humidified atmosphere with 5% CO_2_. Cell viability was assessed by conducting colorimetric measurements of the amount of insoluble formazan formed in living cells based on the reduction of 3-(4,5-dimethyl-thiazol-2-yl)-2,5-diphenyltetrazolium bromide (MTT) (Sigma, St. Louis, MO, USA). Briefly, adherent cells (100 μL) were seeded into each well of a 96-well cell culture plate and allowed to adhere for 12 h before drug addition, while suspended cells were seeded just before drug addition, both with an initial density of 1 × 10^5^ cells/mL in 100 μL of medium. Each tumor cell line was exposed to the test compound at various concentrations in triplicate for 48 h. After the incubation, MTT (100 μg) was added to each well, and the incubation continued for 4 h at 37 °C. The cells lysed with SDS (200 μL) after removal of 100 μL of medium. The optical density of lysate was measured at 595 nm in a 96-well microtiter plate reader (Bio-Rad 680, Hercules, CA, USA). The IC_50_ value of each compound was calculated by Reed and Muench’s method [32].

## 4. Conclusions

A series of novel 4β-triazole-podophyllotoxin glycoconjugates have been synthesized and screened for anticancer activity against a panel of five human cancer cell lines. The majority of the compounds display moderate to weak cytotoxicity against all five cancer cell lines. Among the synthesized compounds, compound **21a** shows the highest potency of anticancer activity, with IC_50_ values ranging from 0.49 to 6.70 μM, which is more potent than the control drug etoposide (**2**). Compound **21a** is derived from D-galactose, having a hydroxyl group at the 4'-postion of the E ring, an α-glycosidic linkage, and no linking spacer between the galactose moiety and the 1,2,3-triazole residue. These findings will be useful for the further research and development of glycosylated podophyllotoxin derivatives as antitumour agents.
